# Somatosensory processing in long COVID fatigue and its relations with physiological and psychological factors

**DOI:** 10.1113/EP093600

**Published:** 2026-09-25

**Authors:** Bethan Thomas, Rachael Pattinson, Geoffrey Cunningham, Christine Bundy, Jennifer L. Davies

**Affiliations:** ^1^ School of Healthcare Sciences Cardiff University Cardiff UK; ^2^ School of Dentistry Cardiff University Cardiff UK

**Keywords:** autonomic nervous system, fatigue, illness perceptions, long COVID, mood, somatosensory system

## Abstract

We aimed to quantify somatosensory processing in long COVID and interactions between somatosensory processing, fatigability, fatigue, autonomic function, mood and illness beliefs. Eighty‐eight participants (44 long COVID and 44 controls) were invited to complete two testing sessions, where fatigue was induced by either a cognitive or a physical task in a cross‐over design; all participants completed at least one session. Baseline questionnaires assessed trait fatigue, autonomic symptoms, mood and illness beliefs. Pre‐ and post‐task measures included somatosensory processing, state fatigue, perceived effort and heart rate variability (HRV). Group differences and task‐related changes were analysed using multivariate and linear mixed models. There was no multivariate group effect on baseline somatosensory measures (*P* = 0.172), nor did they change following exertion or associate with post‐exertion fatigue. Long COVID participants reported greater fatigue than controls, with 64% meeting criteria for severe fatigue. State fatigue was greater at baseline, throughout both exertion tasks (all *P* < 0.001), and increased more during exertion compared with controls. Despite this, cognitive and physical performance changed similarly across tasks, with no group differences in fatigability (*P* = 0.199–0.441). Long COVID participants had lower resting HRV, indicating autonomic dysfunction, but HRV was not associated with fatigue. Only group status (*P* < 0.001) and pre‐task fatigue (*P* < 0.001) were associated with post‐exertion fatigue. Within long COVID participants, greater depression (*P* < 0.001) and perceived illness threat (*P* = 0.033) were associated with greater trait fatigue. The absence of somatosensory abnormalities provides no support for the sensory attenuation model of fatigue in long COVID.

## Abstract (highlights)

- **What is the central question of this study?**
  Is somatosensory processing different between individuals with long COVID fatigue compared to controls?
- **What is the main finding and its importance?**
  The absence of alterations in somatosensory processing provides no support for the sensory attenuation model of fatigue in long COVID. Long COVID participants experienced greater trait and state fatigue compared to controls. State fatigue increased significantly more in long COVID participants during cognitive and physical exertion, but performance did not differ from controls. Findings suggest fatigue is driven by other mechanisms that increase perceived exertion without a decrease in performance.

## 1 INTRODUCTION

Long COVID is a multi‐system condition in which individuals experience persistent symptoms 12 weeks following acute COVID‐19 infection (Crook et al., 2021; NICE, 2020). Fatigue is one of the most common and debilitating symptoms reported in long COVID (Joli et al., 2022), causing a significant impact on individuals, employers and healthcare systems (Townsend et al., 2020).

Fatigue is an experience of symptoms that can only be assessed using self‐report measures. It is a complex phenomenon that is likely to involve overlap of physiological and psychological factors (Matura et al., 2018). Although difficult to define (Kluger et al., 2013), there has been recent consensus within another clinical condition that fatigue is a ‘range of symptoms from mild subjective feelings of tiredness to an overwhelming debilitating, and sustained sense of exhaustion that likely decreases one's ability to execute daily activities and function normally in family or social roles’ (Maxwell et al., 2024). In this study we use a multi‐framework approach to explore the complexity of potential mechanisms and drivers of long COVID fatigue. We specifically focus on the sensory attenuation model of fatigue, the role of autonomic dysfunction, the common‐sense model of self‐regulation, and the mood updating model to explore the complex interactions between physiological and psychological factors in long COVID fatigue.

The sensory attenuation model of fatigue (Kuppuswamy, 2022) proposes that impaired sensory processing increases effort perception, driving fatigue (De Doncker et al., 2018; Kuppuswamy, 2017). Somatosensory deficits exist across clinical conditions that involve fatigue (Jamali et al., 2017; Kessner et al., 2016) and somatosensory processing has been linked to fatigue (Ito et al., 2022). Sensory changes are common in long COVID (Trott et al., 2022), but the sensory attenuation model of fatigue has not been fully explored in this population.

There are two similar, but distinct, phenomena involved in somatosensory processing (Kilteni & Ehrsson, 2022). Attenuation is the suppression of somatosensory input that arises from one's own movement (reafferent input) in comparison to somatosensory input that arises from external sources (exafferent input). Gating is the suppression of externally generated somatosensory input during voluntary movement in comparison to when at rest. Somatosensory gating has been reported not to differ between individuals without long COVID and those reporting long COVID fatigue that had a moderate‐to‐severe impact on daily living (Baker et al., 2023). Previous studies in clinical conditions that experience fatigue have focused on somatosensory attenuation (Parthasharathy et al., 2022; Wolpe et al., 2018), but this has not been studied in long COVID. In this study we aim to replicate the Baker et al. (2023) finding that somatosensory gating is not different in individuals with and without long COVID fatigue, and extend this by asking whether somatosensory attenuation is different between individuals with and without long COVID fatigue (Research Question 1).

Fatigue can be considered as both a stable and an enduring characteristic, reflecting how a person feels over weeks or months, and an instantaneous experience, reflecting how a person feels at a given moment. These concepts are referred to as trait and state fatigue, respectively. Although somatosensory processing has been studied in relation to trait fatigue (Baker et al., 2023; De Doncker et al., 2021), it is not well studied in relation to state fatigue. Definitions and measures of fatigue in long COVID cover different dimensions, such as physical, cognitive, emotional, psychosocial and general fatigue as well as post‐exertional malaise (Thomas, Pattinson, Edwards et al., 2024). It is unclear if different dimensions share the same underlying mechanisms. Here we focus on state fatigue in two of these dimensions – physical and cognitive. We will quantify relations between somatosensory processing and state fatigue induced by both a cognitive and a physical task (Research Question 2) and quantify how these are related to trait fatigue (Research Question 4). This will add to our understanding of the relations between and mechanisms underlying different dimensions of state fatigue and trait fatigue in long COVID.

The sensation of fatigue may be independent from objective measures of performance (Bailey et al., 2007; Krupp & Elkins, 2000; Lou et al., 2003). For example, Fietsam et al. (2023) found that individuals with long COVID reported more fatigue than individuals without long COVID but had similar levels of performance decline on an isokinetic fatigue task. The decline in an objective measure of performance over a discreate period of time is termed performance fatigability (Enoka et al., 2021). Understanding the association between fatigue and fatigability has been identified as an important goal for clinical research (Kluger et al., 2013). We will address this in our second research question by quantifying whether the change in performance over the cognitive and physical tasks (performance fatigability) is related to state fatigue induced by that task, whether this moderates the relation between somatosensory processing and state fatigue, and whether this is impacted by long COVID (Research Question 2).

**FIGURE 1:**
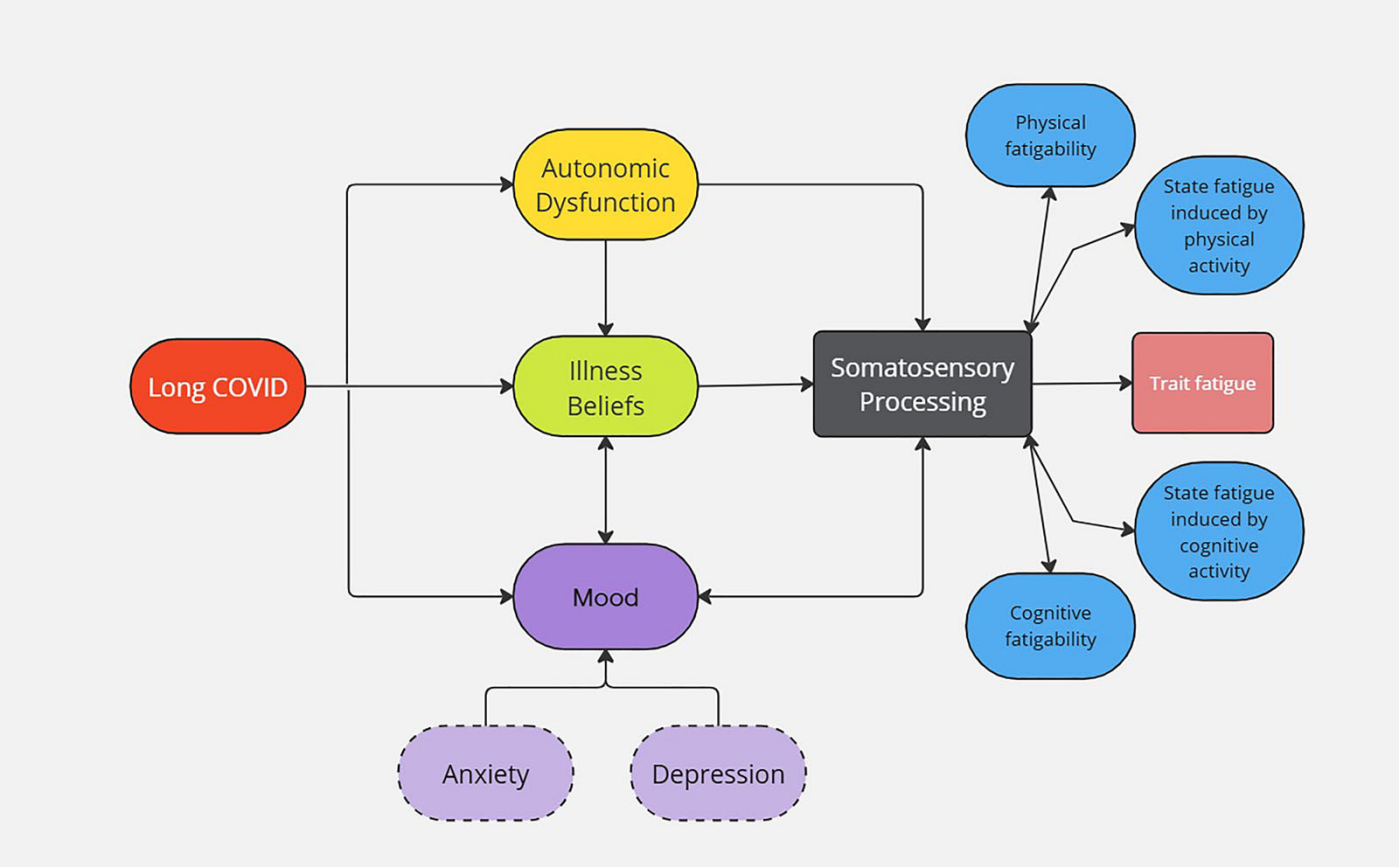
Concept of interactions. Schematic diagram indicating the proposed relations between autonomic dysfunction, illness beliefs, mood (anxiety and depression) and somatosensory processing. We postulate that each of these factors (autonomic dysfunction, illness beliefs, mood) will impact fatigue via an impact on somatosensory processing. We also propose that fatigability and acute (state) changes in fatigue will have a bidirectional relation with somatosensory processing.

In addition to understanding if somatosensory processing predicts fatigue, it is also of relevance to ask if fatigue impacts somatosensory processing (bidirectional arrows in Figure 1, blue boxes). This would potentially indicate that activities that increase fatigue and cause performance fatigability could lead to disrupted somatosensory processing, further increasing the fatigue and fatigability. This will be addressed in our third research question, where we ask if the change in somatosensory attenuation (Research Question 3a) and somatosensory gating (Research Question 3b) from pre‐ to post‐task is predicted by the change in state fatigue from pre‐ to post‐task, fatigability during the task, population (long COVID/controls) and task (cognitive/physical).

In conditions such as depression, autonomic dysfunction is a mediating factor in the experience of fatigue (Costa et al., 2023). Autonomic dysfunction is prevalent in long COVID (Dotan et al., 2022), potentially reflecting a disrupted balance between sympathetic and parasympathetic nervous systems (Jammoul et al., 2023). However, this has not yet been directly related to fatigue in this population. We propose that autonomic dysfunction may impact fatigue via an effect on somatosensory processing (Figure 1). No studies across any clinical conditions have explored the relations between autonomic dysfunction, somatosensory processing and fatigue. This will be considered in Research Question 4 where we explore the role of the autonomic nervous system in trait fatigue. Additionally in exploratory analysis we will look at: (i) the effect of autonomic dysfunction on state fatigue via an effect on somatosensory processing, and (ii) the relations between autonomic dysfunction during exertion, state fatigue during exertion and perceived effort during exertion.

The common‐sense model of self‐regulation (Leventhal et al., 2016) proposes that how an individual perceives their symptoms can be influenced by their emotional responses, previous experiences and beliefs. The mood updating model proposes that prior beliefs about the signals resulting from an action, as well as mood (particularly depression and anxiety), can impact perception of and response to stimuli (Clark & Watson, 2023). Depression and anxiety are a feature of long COVID (Fancourt et al., 2023), and the experience of long COVID symptoms has a profound impact on self‐identity and beliefs about illness (Callan et al., 2022). There is likely a complex interaction between illness beliefs, depression and anxiety, perception of stimuli, and symptom experience in long COVID that has not yet been explored. Understanding individual experiences driving symptom perception and response is essential for targeted interventions. These relations will be explored in Research Question 4, where we quantify if among individuals with long COVID, if trait fatigue is affected by the increase in state fatigue induced by a physical task, the increase in state fatigue induced by a cognitive task, mood, illness beliefs, autonomic symptoms and autonomic nervous system function.

### 1.1 Research questions

Research Question 1. Is pre‐task (baseline) somatosensory attenuation (Research Question 1a) and somatosensory gating (Research Question 1b) different between individuals with long COVID fatigue compared to controls?
Research Question 2. Is state fatigue following exertion predicted by somatosensory attenuation at baseline, somatosensory gating at baseline, the presence of long COVID (long COVID/controls) and the type of task (cognitive/physical), and is performance fatigability a moderator variable in this relation? State fatigue prior to exertion will be included as a covariate in this analysis.
Research Question 3. Is the change in somatosensory attenuation (Research Question 3a) and somatosensory gating (Research Question 3b) from pre‐ to post‐task predicted by the change in state fatigue during exertion, fatigability, the presence of long COVID (long COVID /controls) and the type of task (cognitive/physical)?
Research Question 4. Among individuals with long COVID, is trait fatigue predicted by the increase in state fatigue induced by a physical task, the increase in state fatigue induced by a cognitive task, mood, illness beliefs, autonomic symptoms and autonomic nervous system function?

This study provides the first comprehensive investigation into the role of somatosensory processing in long COVID fatigue and explores how prominent symptoms in long COVID (autonomic dysfunction, altered mood, beliefs about the meaning of symptoms) interact with somatosensory processing, fatigability and the experience of fatigue.

## 2 METHODS

### 2.1 Ethical approval

Prior to recruitment of pilot participants, this study received ethical approval from Cardiff University School of Healthcare Sciences Research Ethics Committee (REC1160). The study conforms to the standards set by the *Declaration of Helsinki*, except for being registered in a publicly accessible database. This study conducted experiments on humans and written informed consent was obtained from all participants.

### 2.2 Participants and recruitment

Participants were recruited via convenience sampling from the local community using passive recruitment methods (digital and physical posters and announcements). Recruitment was targeted to match long COVID and control groups for age and sex. To be eligible, individuals had to be between 18 and 69 years old. Somatosensory attenuation and gating change with age (Parthasharathy et al., 2022), particularly after the age of 69 years (Timar et al., 2023). This age group was therefore excluded, to prevent age‐related effects on somatosensory attenuation and gating confounding the results. Participants had to be able to speak and understand English and be able to provide informed consent. Participants were excluded if they had any physical or psychiatric condition that would prohibit them from walking safely on a treadmill, taking part in a cognitive task, or completing the full battery of assessments (Supplementary material 1).

For inclusion in the control group, individuals may have previously tested positive for COVID‐19 but must have been clear of all symptoms by 12 weeks following acute infection and must not report any ongoing fatigue. Participants in the control group, must not have reported any underlying condition that interferes with their daily function. All inclusion and exclusion criteria were evaluated by self‐report measurements (Supplementary material 1).

For inclusion in the long COVID group, individuals had to self‐report a confirmed COVID infection (confirmed by positive lateral flow test or PCR test, or a clinical diagnosis) and report signs and symptoms that continued or developed 12 weeks or more after acute COVID‐19 infection (NICE, 2020). As this study focused on the impact of fatigue, individuals had to report fatigue (physical, cognitive, mental, emotional, psychosocial fatigue or post‐exertional symptom exacerbation) as one of their long COVID symptoms and must not have experienced any form of ongoing fatigue prior to COVID infection (Supplementary material 1).

### 2.3 Experimental procedures

Prospective participants were provided with a participant information sheet and an opportunity to ask questions. If they wished to participate, they were directed to an online platform (JISC) and asked to fill in a consent form. Once informed consent had been provided, the participant was contacted to schedule the two testing sessions.

**FIGURE 2:**
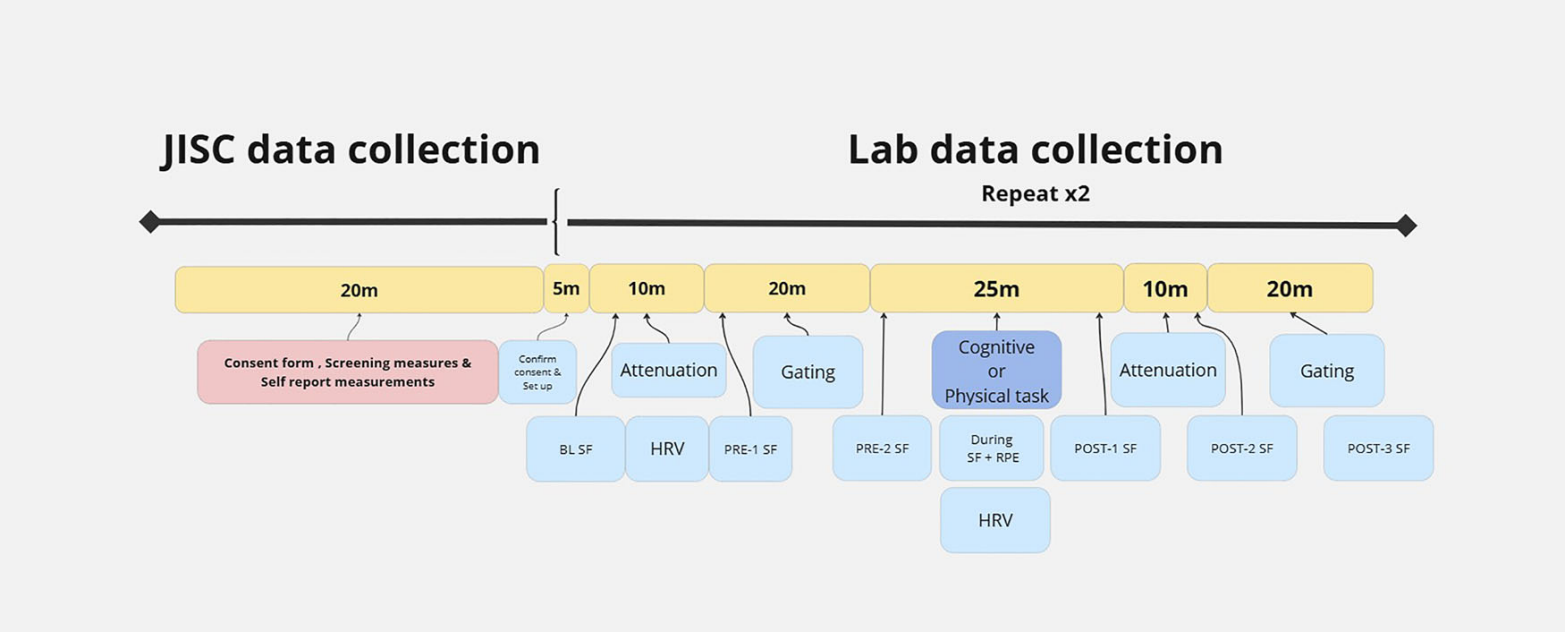
Data collection timeline. JISC is a digital platform designed specifically for education and research organisations, that can be used for the creation and dissemination of surveys. BL, baseline; PRE‐1, immediately after pre‐task somatosensory attenuation measure; PRE‐2, immediately after pre‐task somatosensory gating measure; POST‐1, immediately after the completion of the task (cognitive or physical); POST‐2, immediately after post‐task somatosensory attenuation measure; POST‐3, immediately after post‐task somatosensory gating measure; HRV, heart rate variability.

A schematic illustration of the study timeline is shown in Figure 2. Prior to the testing sessions, participants were directed to the online platform (JISC) which they were able to access from any place with internet connection to provide self‐reported measurements (Supplementary material 1) and complete a battery of validated measures (see outcome measures). A contact email for the research team was provided on every page of the online platform, in case of questions. Participants were able to pause and restart this process multiple times. Pilot tests indicated that this process took 60 min in total.

On completion of the participant characteristics and validated measures, participants were given a unique, randomly generated four‐digit identifier. Consent forms, which contained the participants personal details, were saved separately to all other data only identified with the participants four‐digit identifier.

The protocol was split into two testing sessions. Each session took up to 90 min. The two sessions were identical, except that in one session participants performed a cognitive task and in the other session they performed a physical task. Participants were asked to complete both sessions in a cross over design. In each session, somatosensory processing (attenuation and gating) was measured pre‐ and post‐task (physical or cognitive). The order of the two sessions was randomised using the command randi([1 2]) in MATLAB software (version 23.2, MathWorks Inc., Natick, MA, USA), which was run on the morning of the first testing session. A value of ‘1’ indicated that cognitive task would be performed first and ‘2’ indicates that the physical task would be performed first. Participants were asked to return on a separate day for each session.

Upon arrival at each testing session, the participant was re‐consented to ensure that they were still happy to participate and then prepared for data collection. The measure of somatosensory gating requires activity of the first dorsal interosseus muscle to be monitored in real time (see outcome measures). To achieve this, the skin over the first dorsal interosseus muscle was prepared using a mild abrasive gel (NuPrep, Weaver and company, Aurora, CO, USA) and a Trigno Quattro Sensor (Delsys Europe, Manchester, UK) was placed over the muscle belly parallel to the radial border of the second metacarpal, just proximal to the junction between the muscle and distal tendon (Zijdewind et al., 1995). The reference sensor was placed on the forearm. Heart rate was pre‐registered to be measured using a Polar H10 Hartslagsensor (Polar Electro, Leamington Spa, UK) streamed into Dflow software (Motek Medical B.V., CL Houten, The Netherlands). However, this equipment became unavailable. Heart rate was therefore measured using an Trigno Avanti sensor (Delsys) placed on the sternum and streamed into Vicon Nexus software (Oxfrod, UK).

Pre‐task measures (somatosensory gating, somatosensory attenuation, state fatigue) were evaluated (see Outcome measures and Figure 2). Following this, the participant was asked to perform the task (cognitive or physical, depending on the session). Immediately following the task, post‐task measures (somatosensory gating, somatosensory attenuation, state fatigue) were evaluated (see Outcome measures and Figure 2).

#### 2.3.1 Cognitive task

The cognitive task was based on that developed by Hassan et al. (2024). It consists of a battery of four different cognitive tasks, each designed to challenge a different aspect of executive function: A‐X Continuous Performance Test (CPT), n‐back, mental rotation task and visual search task. The AX‐CPT was chosen as the task in which to measure task performance, and therefore it was presented first and last as in Hassan et al. (2024). The remaining tasks were presented in a pseudorandom order as designed in Hassan et al. (2024), where the tasks did not repeat back‐to‐back; the time between tasks being repeated was maximised; the time between tasks being repeated was similar between the different tasks, so that if any order effect was present, it would be the same for all participants.

In the original study Hassan et al. (2024), each cognitive task was performed for 10 min at a time and was repeated three times, resulting in a total duration of 2 h (four tasks × 10 min per task × three repeats = 4 × 10 min × 3 = 120 min). In the present study we reduced the duration of each task to 2 min, giving a total duration of 24 mins (four tasks × 2 min per task × three repeats = 4 × 2 min × 3 = 24 min). This was done in consultation with our patient and public involvement group to avoid placing excessive burden on a population that reports high fatigue levels. The modified duration was expected to induce fatigue as participants are required to task switch between multiple short‐duration tasks, constituting an additional demand that increases cognitive fatigue (Dang et al., 2013). Testing of this modified task in 15 young healthy individuals indicated a significant increase in fatigue from pre‐ to post‐task. Fatigue measured on a 100‐mm visual analogue scale (VAS) increased from a mean (standard error) of 26 (5) mm pre‐task to 59 (5) mm post‐task (Student's paired *t*‐test, *t* = −7.251; *P* < .001). Fatigue measured using the fatigue subscale of the Brunel Mood Scale increased from a median (interquartile range) of 7.0 (1.5) pre‐task to 9.0 (4.0) post‐task (Wilcoxon signed rank test, *Z* = 2.991; *P* = 0.003) (Corfield and Davies, unpublished data, in Thomas, Pattinson, Bundy et al. (2024). Participants were sent a training version of this protocol prior to attending the lab session, this allowed them to familiarise themselves with the tasks involved to minimise any learning effect during the testing session. The cognitive task was completed on a computer that was placed in front of participants. Full details of the task are in Supplementary material 2.

#### 2.3.2 Physical task

The physical task was the 6‐min walk task (6MWT). This required participants to walk at their fastest pace for 6 min, aiming to cover as much ground as possible. Participants were able to take breaks at any point, with the clock continuously running (American Thoracic Society, 2002).

The test was carried out on a self‐paced treadmill (Motek Medical B.V.). Four retroreflective markers were placed on the pelvis over the left and right anterior and posterior superior iliac spines. The position of these markers was recorded by a three‐dimensional optoelectronic motion capture system (Vicon Nexus) and streamed in real time into D‐flow software version 3.36.2 (Motek Medical B.V.). D‐flow software monitored the position of these markers and controlled treadmill speed according to the position of the participant. This allowed the participant to walk on the treadmill at a fluctuating speed, including slowing to a stop for rests whenever they want. The speed of the treadmill belt and the distance covered was recorded within D‐flow software. Participants were fitted with a security harness and were allowed to familiarise themselves with the self‐paced treadmill for up to 5 min prior to the start of the physical task. Participants were allowed to hold onto the siderails for stability.

The 6MWT induced fatigue in individuals with multiple sclerosis (McLoughlin et al., 2016), indicating it is an appropriate task to induce fatigue in long COVID participants. It is not anticipated that this task will cause significant fatigue in healthy (control) participants. However, healthy (control) participants were asked to perform the task to provide control data and inform interpretation of data from participants with long COVID fatigue.

### 2.4 Outcome measures

#### 2.4.1 Participant characteristics

Participant characteristics (age, sex, ethnicity) were captured using a custom form (Supplementary material 1). Trait fatigue (severity and impact), emotional symptoms, illness beliefs and autonomic nervous system symptoms were evaluated using the validated measures outlined below. This allowed appropriate descriptive reporting of the characteristics of the study sample and also was used in exploratory analyses to develop future hypotheses (Research Question 4).

The severity of trait fatigue was evaluated using the Fatigue Assessment Scale (FAS) and the Chalder Fatigue Questionnaire (CFQ‐11). The impact of fatigue was evaluated using the Modified Fatigue Impact Scale (MFIS). The FAS is a validated unidimensional measure of fatigue that evaluates fatigue severity (Michielsen et al., 2003). The FAS consists of 10 items, each on a 5‐point rating scale, with total score ranging from 10 to 50. The FAS provides one unidimensional score for fatigue, with higher scores indicating greater fatigue severity. This measure is recommended as a core outcome measure for long COVID fatigue (Gorst et al., 2023) and inclusion allows for comparison across long COVID research. The CFQ‐11 (Chalder et al., 1993) and MFIS (Ritvo et al., 1997) are multidimensional measures that provide individual scores for individual dimensions of fatigue. The CFQ‐11 consists of 11 items, each on a three‐point Likert scale. The CFQ‐11 produces two individual scores of mental and physical fatigue. The MFIS consists of 21 items, which provide three individual scores of the perceived impact of physical, cognitive and psychosocial fatigue.

Self‐reported autonomic system function was evaluated using the Composite Autonomic Symptom Score‐31 (COMPASS‐31) scale, which evaluates neurodegenerative system symptoms through 31 patient‐reported questions (Sletten et al., 2012). Assessment is through six weighted domains: orthostatic intolerance (10 points), vasomotor (6 points), secretomotor (7 points), gastrointestinal (28 points), bladder (9 points) and pupillomotor (15 points). A higher score indicates higher severity of autonomic dysfunction. The COMPASS‐31 has been used to measure autonomic dysfunction in long COVID populations. A large survey of individuals with long COVID found that 66% of patients were classified as having moderate to severe autonomic dysfunction quantified as a COMPASS‐31 score of ≥20 (Larsen et al., 2022).

Mood was evaluated using the Hospital Anxiety and Depression Scale (HADS) (Zigmond & Snaith, 1983), which is a reliable and valid measure for assessing anxiety and depression (Herrmann, 1997). The HADS comprises 14 items, seven relating to anxiety and seven to depression. Items are rated on a four‐point rating scale and produce two scores, one for anxiety (HADS‐A) and one for depression (HADS‐D). Scores can be interpreted as non‐cases (<7), mild (8–10), moderate (11–14) and severe symptoms (15–21) (Zigmond & Snaith, 1983).

Illness beliefs were evaluated using the Brief illness perception questionnaire (B‐IPQ) (Broadbent et al., 2006). The B‐IPQ is an eight‐item questionnaire that assesses cognitive illness representations, emotional illness representations and illness coherence representation. The B‐IPQ has evidence of test–retest reliability and concurrent and discriminant validity (Broadbent et al., 2006). Each item was rated on a 0–10 scale, with higher scores indicating a more threatening perception of the illness. The total score was calculated by summing the scores of all eight items, with a possible range of 0–80. Higher scores indicate distorted or unhelpful illness beliefs.

#### 2.4.2 State fatigue

State fatigue was evaluated using a 100‐mm VAS that asked participants to rate their current level of fatigue. The VAS was anchored on the left side with ‘not fatigued at all’ and on the right side with ‘extremely fatigued’ (Supplementary material 3).

Participants were asked to make a line on a paper copy of the VAS at six time points across each testing session: at baseline (BL), immediately after the pre‐task somatosensory gating measure (PRE‐1), immediately after the pre‐task somatosensory attenuation measure (PRE‐2), immediately after the completion of the task (cognitive or physical) (POST‐1), immediately after the post‐task somatosensory gating measure (POST‐2), and immediately after the post‐task somatosensory attenuation measure (POST‐3) (Table 1 and Figure 2). This was to allow evaluation of the time course of fatigue and recovery of fatigue over the period required for the outcome measures. State fatigue at each time point was quantified as the distance of the mark made by the participant from the left‐edge of the horizontal line (range 0–100 mm).

**TABLE 1:** Outcome measures.

		Time point of measurement		
Concept	Measure	Prior to first testing session	Cognitive task testing session	Physical task testing session
Severity of trait fatigue	FAS CFQ‐11 total CFQ‐11 physical subscale CFQ‐11 mental subscale	Prior to session		
Impact of trait fatigue	MFIS physical subscale MFIS cognitive subscale MFIS psychosocial subscale	Prior to session		
Autonomic symptoms	COMPASS‐31	Prior to session		
Mood	HADS‐A HADS‐D	Prior to session		
Illness beliefs	BIPQ	Prior to session		
Autonomic function	HRV		During somatosensory attenuation measures and throughout cognitive task	During somatosensory attenuation measures and throughout physical task (6MWT)
State fatigue	Visual analogue scale		BL, PRE‐1, PRE‐2, POST‐1, POST‐2, POST‐3	BL, PRE‐1, PRE‐2, POST‐1, POST‐2, POST‐3
State fatigue	Numeric rating scale		After every fourth task and at the end of the cognitive task	At 90 s, 180 s and 270 s after the start of the 6MWT and at the end of the 6MWT
Perceived effort	Rating of perceived exertion scale		After every fourth task and at the end of the cognitive task	At 90 s, 180 s and 270 s after the start of the 6MWT and at the end of the 6MWT
Cognitive fatigability	BIS		During cognitive task	
Physical fatigability	Average walking speed from 330 to 360 s of 6MWT minus average walking speed from 30 s to 60 s			During 6MWT task
Somatosensory attenuation	Mean force overcompensation at each target force Intercept and slope from a linear regression of matched versus target force		Pre‐ and post‐cognitive task	Pre‐ and post‐physical task (6MWT)
Somatosensory gating	*I*_50_rest_/*I*_50_movement_		Pre‐ and post‐cognitive task	Pre‐ and post‐physical task (6MWT)

Abbreviations: BIPQ, Brief Illness Perception Questionnaire; BIS, Balanced Integration Score; BL, baseline; CFQ‐11, Chalder Fatigue Questionnaire; COMPASS‐31, Composite Autonomic Symptom Score; FAS, Fatigue Assessment Scale; HADS‐A, Hospital Anxiety and Depression Scale anxiety subscale; HADS‐D, Hospital Anxiety and Depression Scale depression subscale; HRV, heart rate variability; MFIS, Modified Fatigue Impact Scale; POST‐1, immediately after the completion of the task (cognitive or physical); POST‐2, immediately after the post‐task somatosensory attenuation measure; POST‐3, immediately after the post‐task somatosensory gating measure; PRE‐1, immediately after pre‐task somatosensory gating measure; PRE‐2, immediately after pre‐task somatosensory attenuation measure.

In addition, state fatigue was reported using a numerical rating scale (NRS) at four time points during the physical and cognitive tasks. This was at 90 s, 180 s and 270 s of the 6MWT and after every fourth task in the cognitive task and at the end of each task. In the cognitive task these ratings were time stamped. Participants were shown the NRS, which consists of a scale from 0 to 10, with 0 being anchored as ‘not fatigued at all’ and 10 being anchored as ‘extremely fatigued’. They were asked to verbally report the number that corresponds to their fatigue level. The need to switch from a VAS to an NRS is dictated by the inability of participants to physically mark a line on a piece of paper while completing the 6MWT.

#### 2.4.3 Perceived effort

Perceived effort was evaluated using the Borg rating of perceived exertion (RPE) scale. This requires participants to rate how much effort an activity takes on a scale of 6 (no exertion at all) to 20 (maximal exertion). RPE was measured at the same time points as the NRS. Participants were shown the RPE scale prior to carrying out the tasks, as done in previous studies where participants are asked to report their RPE during a task (Flairty & Scheadler, 2020). This allowed us to make distinctions between perceived effort and fatigue during exertion. Participants were shown the RPE scale during each task, and were asked to verbally report the number that corresponded to their level of exertion.

#### 2.4.4 Autonomic nervous system function

Autonomic nervous system function was quantified using heart rate variability (HRV), calculated as the proportion of successive intervals which differ by >50 ms (Baker et al., 2023). Heart rate was pre‐registered to be measured using a Polar H10 Hartslagsensor (Polar, UK) streamed into Dflow software (Motek Medical B.V.). However, this equipment became unavailable. Heart rate was therefore measured using an Trigno Avanti sensor (Delsys) placed on the sternum and streamed into Vicon Nexus software. HRV was measured during the somatosensory attenuation measure to ensure that participants are sitting quietly, not engaged in trial activity. This will replicate previous measurement in individuals with long COVID to allow comparison (Baker et al., 2023). HRV was additionally measured throughout the cognitive and physical tasks to allow exploratory analysis of the change in autonomic nervous system function during exertion. HRV measured over a short period mostly reflects parasympathetic nervous system activity (Gullett et al., 2023). HRV has been used as a measure of autonomic nervous system function to quantify autonomic function changes in response to various interventions (Ali & Chen, 2023). The code used to calculate HRV is provided in Supplementary material 4.

#### 2.4.5 Fatigability during the cognitive task

Fatigability during the cognitive task (Research Question 2) was quantified as reported by Hassan et al. (2024) using the balanced integration score (BIS) (Liesefeld & Janczyk, 2019). The AX‐CPT was chosen as the task in which to measure task performance, and therefore it was presented first and last in the battery of tasks, following the protocol of Hassan et al. (2024). The BIS was calculated using the reaction time and response data during the first and last repeat of the AX‐CPT task. The BIS combines reaction time and accuracy into a single metric which is standardised across all time points and participants. A BIS score of zero represents an average level of performance across all participants and conditions, with above average and below average performance indicated by positive and negative numbers, respectively. The code used to calculate the outcome measure is available at https://github.com/Liesefeld/BIS.

#### 2.4.6 Fatigability during the physical task

Fatigability during the 6MWT (Research Question 2) was quantified as the change in average walking speed (m s^−1^) from the beginning (30–60 s) to the end (330–360 s) of the test (Andersen et al., 2016; Witherspoon et al., 2018). The first time point was taken after the initial 30 s to avoid confounding from acceleration. Example data are shown in Thomas, Pattinson, Bundy et al. (2024) and the code used to calculate fatigability during the 6MWT is available in Supplementary material 5.

#### 2.4.7 Somatosensory processing

Two measures of somatosensory processing were evaluated at each of the pre‐ and post‐task time points: a measure of somatosensory attenuation and a measure of somatosensory gating.

#### 2.4.8 Somatosensory attenuation

The somatosensory attenuation assessment took approximately 10 mins. Participants were seated comfortably and positioned with their dominant hand placed on a table with their palm facing upwards. Participants were given a practice trial to make sure they understand the task.

An audible beep marked the start of the trial. The researcher (B.T.) pressed on the abductor pollicis brevis muscle of the dominant hand with a Digital Handheld Dynamometer (microFET2; Hoggan Scientific, Salt Lake City, UT, USA). The force was applied through a digit transducer pad of 1 cm diameter to a magnitude of one of 4, 5, 6 or 7 N, and was maintained for 3 s until a second beep marks the end of the 3 s. This constitutes an exafferent force. The magnitude of force applied was displayed in real time on the device and was visible to the researcher, but not to the participant. After 2 s rest, a third beep sounded, and the participant was asked to press an identical probe onto the same section of skin with the same force that was exerted by the researcher. This constitutes a reafferent force (Kilteni & Ehrsson, 2022).

Following a practice trial, each force level (4, 5, 6 or 7 N) was performed eight times, for a total of 32 trials. These trials were organised in two blocks of 16 trails. The force level was randomised within MATLAB software. There was an interval of 60 s between the two blocks. The average force across the 3 s was stored in the dynamometer for each trial and was retrieved after the end of the session.

Somatosensory attenuation was quantified as the mean force overcompensation between the exafferent force (generated by the researcher) and the reafferent force (generated by the participant). Mean force overcompensation was calculated as the average difference between the exafferent and reafferent force (Wolpe et al., 2018), on each trial (*n* = 8) across each force level (*n* = 4). To examine force matching as a function of force level, the intercept and slope from the linear regression between exafferent and reafferent force was calculated for each participant and condition. Example data is shown in Thomas, Pattinson, Bundy et al. (2024) and the code used to calculate the outcome measure is provided in Supplementary material 6.

#### 2.4.9 Somatosensory gating

The protocol to evaluate somatosensory gating replicated that of Baker et al. (2023) to allow direct comparison of results and took approximately 20 min. The participant was seated comfortably and positioned with their dominant hand placed on a table.

The index finger of the dominant hand was stimulated with single, constant current, square wave pulses of 2‐ms duration by a constant current stimulator (model DS7A; Digitimer; Welwyn Garden City, UK) connected to adhesive surface electrodes (71505‐K/C/12; Ambu Limited, Huntingdon, UK) placed on the proximal and middle phalanges.

Stimulation was mild, starting below perceptual threshold and rising to the threshold at which it could just be perceived, but never above this. At this threshold, the stimulation feels like a mild transient ‘tingle’. Perceptual threshold was determined prior to the task by asking participants to report verbally when they felt a stimulation on their index finger. The initial stimulus intensity was 0.10 mA. Based on previous work (Chapman et al., 1987), we expected this to be imperceptible to all participants. The intensity was adjusted using an ascending–descending–ascending staircase method as follows:

- A set of up to six stimuli were delivered at the initial intensity. If the participant did not report perceiving a stimulus on at least three out of six trials, stimulation intensity was increased by 0.10 mA for the next set. This continued until the participant reported that they perceived a stimulus on at least three out of six trials.
- At this point, the stimulus intensity was then decreased by 0.05 mA per set until the participant did not report perceiving a stimulus on three out of six trials.
- The stimulation intensity was then increased by 0.01 mA per set until the participant again reported that they perceived a stimulus on three out of six trials. This value was recorded as the perceptual threshold.

Based on previous work (Baker et al., 2023; Chapman et al., 1987), it was anticipated that perceptual threshold would range from 0.23 to 1.11 mA across participants. The thresholds in this study ranged from 0.11 to 1.93 mA.

One hundred trials were performed, during which participants were given a stimulus (*P* = 0.8) or not (*P* = 0.2). Fifty of these were ‘resting’ trials, and 50 were movement trials. In a resting trial, participants were asked if they were ready, and asked to report if they felt a stimulation or not. In a movement trial, participants were asked if they were ready, and then instructed to make a rapid index finger abduction movement and report if they felt a stimulation or not. Stimulation was delivered in 80% of the trials. Activity of the first dorsal interosseus muscle was monitored using surface electromyography (Delsys Trigno Quattro Sensor) and monitored in real time in D‐flow software (Motek Medical B.V.). The software was programmed to detect when the muscle activity started to increase above baseline and delivered a stimulus (*P* = 0.8) or not (*P* = 0.2) 50 ms after this onset of muscle activity. In this way, the timing of the stimulus in the movement trials was directly linked to movement onset. In all trials, participants were given up to 5 s to respond if they felt the stimulus or not. If they were unable to respond, this was reported as stimulus not perceived.

Rest and movement trails (*n* = 50 of each) were randomised. Stimulus intensity started at the pre‐determined perceptual threshold and was subsequently controlled in equal steps separately for rest and movement trials (Baker et al., 2023). If the participant perceived the stimulus, it decreased by 0.02 mA for the next trial in that condition. If the participant did not perceive the stimulus, it increased by 0.02 mA for the next trial in that condition. There was an inter‐stimulus interval of at least 3 s between consecutive trials.

Somatosensory gating was quantified in the same way as reported by (Baker et al., 2023) using code provided by these authors. The probability of detection at intensity I was fitted to a sigmoid curve where *I*_50_ is the intensity with 50% detection, and *k* determines the slope of the curve:

*P*(Detection at intensity *I*|*I*_50_, *k*)* =* $\frac{1}{1+\exp[(I_{50}-I)/k]}$. This was quantified in rest and movement trials, and somatosensory gating as *I*_50_movement_/*I*_50_rest_. Example sigmoid curves calculated at rest and during movement are shown in Figure 4 in Thomas, Pattinson, Bundy et al. (2024).

(In the Stage 1 Registered Report we stated that we would quantify somatosensory gating ‘in the same way as reported by Baker et al. (2023) … *I*_50_movement_ – *I*_50_rest_’. This was a typographical error, as Baker et al. used the formula *I*_50_movement_/*I*_50_rest_. This is corrected here.)

### 2.5 Data inclusion and exclusion

It was preregistered that participants failing to complete the cognitive task would be excluded from the analysis for Research Question 2, 3 and 4 as we would not be able to compare the impact of task. However, their data would be included in other exploratory analysis where the cognitive task was not included as a predictor. It was preregistered that participants who did not complete the post‐task measures in either session, due to withdrawing from the study, would be excluded from the analysis for Research Question 3. Two sessions (one cognitive, one physical) were stopped early at the participant's request; both were individuals with long COVID, and their data was included in analysis where the post‐task measure was not included as a predictor.

### 2.6 Data analysis

Participant characteristics (age, sex, ethnicity) are described using measures of frequency distribution. All other outcomes (FAS score, CFQ‐11 total and subscale scores, MFIS total and subscale scores, COMPASS‐31 score, HADS score, B‐IPQ score, BIS score, change in walking speed, HRV during quiet sitting in each session, HRV throughout each task (physical, cognitive), VAS score at six time points in each session, NRS at four time points during each task, RPE at four time points during each task, somatosensory attenuation pre‐ and post‐task in each session, somatosensory gating pre‐ and post‐task in each session) are described using measures of central tendency and distribution. A list of all outcomes is provided in Table 1.

### 2.7 Statistical analysis

A series of statistical analyses were conducted to compare the descriptive characteristics of the two groups (long COVID/neither long COVID nor fatigue). The characteristics compared were age, sex, trait fatigue (FAS score, CFQ‐11 total score, CFQ‐11 physical subscale score, CFQ‐11 mental subscale score), impact of trait fatigue (MFIS physical subscale score, MFIS cognitive subscale score, MFIS psychosocial subscale score), Mood (HADS‐A score, HADS‐D score), illness beliefs (BIPQ score), and autonomic symptoms (COMPASS‐31 score). Sex was analysed as a categorical variable. A chi‐square test was used to compare the sex distribution between the groups. All other variables were analysed as ratio data and compared across groups using an independent samples Student's *t*‐test if the data met parametric assumptions (normality, verifying equal variance) and a Mann–Whitney *U*‐test if the data did not meet parametric assumptions.

Results are summarised using a measure of central tendency (mean or median) and spread (standard deviation or interquartile range), test statistics, and *P*‐values for ratio data and frequency, percentage, chi‐square values and *P*‐values for categorical variables.

Hypothesis 1 Pre‐task (baseline) somatosensory attenuation will be different between individuals with long COVID fatigue and controls. We also preregistered an attempt to replicate the Baker et al. (2023) finding that somatosensory gating is not different between individuals with long COVID fatigue and controls. The independent variable was ‘participants’, which consists of two categories: ‘individuals with long COVID fatigue’ and ‘controls’. The two dependent variables were ‘somatosensory attenuation’ and ‘somatosensory gating’, measured at baseline during the first session participants attend.

This was tested using a multivariate analysis of variance to test for an overall difference in somatosensory processing between populations, and to explore the differences in somatosensory gating and attenuation. We checked that the following assumptions were met: multivariate normality, independence and equal variance. If the multivariate analysis of variance was significant, we planned to conduct follow‐up univariate analysis of variance for somatosensory attenuation and somatosensory gating to identify specific contributions to the effect and planned to apply Bonferroni correction to control for the increased risk of Type I error.

Hypothesis 2 : State fatigue following exertion will be predicted by somatosensory attenuation at baseline, somatosensory gating at baseline, the presence of long COVID (long COVID/controls) and the type of task (cognitive/physical). Performance fatigability is considered a moderator variable in these relations. State fatigue prior to exertion is included as a covariate in this analysis.

This was tested using a linear mixed model:

$$Y_{ijk}=\mu +a_{i}+b_{j}+c_{k}+d_{ik}+e_{ik}+f_{ik}+g_{ijk}+\left(f_{ik}\times a_{i}\right)+\left(f_{ik}\times b_{j}\right)+\varepsilon_{ijk}$$

where:

- *Y_ijk_* is state fatigue following exertion in the *i*‐th group (long COVID/control) during task *j* (cognitive/physical) for the *k*‐th subject.
- μ is the overall mean of state fatigue following exertion.
- *a_i_* is the effect of the *i*‐th group (fixed effect: long COVID vs. control).
- *b_j_* is the effect of the *j*‐th task (fixed effect: cognitive vs. physical).
- *d_ik_* is the baseline somatosensory attenuation for the *k*‐th subject in the *i*‐th group.
- *e_ik_* is the baseline somatosensory gating for the *k*‐th subject in the *i*‐th group.
- *f_ijk_* is the performance fatigability for the *k*‐th subject in the *i*‐th group during the *j*‐th task.
- *g_ijk_* is the state fatigue prior to exertion for the *k*‐th subject in the *i‐*th group during the *j*‐th task (covariate).
- (*f_ik_* ×*a_i_*) is the interaction term between performance fatigability and group.
- (*f_ik_* ×*b_j_*) is the interaction term between performance fatigability and task.
- ϵ*_ijk_* is the residual error term, assumed to be normally distributed.

Hypothesis 3 The change in somatosensory attenuation (Research Question 3a) and somatosensory gating (Research Question 3b) from pre‐ to post‐task will be predicted by the change in state fatigue from pre‐ to post, fatigability, the presence of long COVID (long COVID/controls), and the type of task (cognitive/physical).

We used linear mixed models to test these hypotheses, with separate models for somatosensory attenuation and somatosensory gating:

$$Y_{ijk}=a_{i}+b_{j}+c_{ijk}+d_{ijk}+u_{0i}+\varepsilon_{ijk}$$

where:

- *Y_ijk_* ​: Represents attenuation or gating at baseline in the *i*‐th group (long COVID/control) during task *j* (cognitive/physical) for the *k*‐th subject.
- *a_i_​*: The fixed effect of the *i*‐th group (long COVID vs. control) on baseline attenuation.
- *b_j_​*: The fixed effect of the *j*‐th task (cognitive vs. physical) on baseline attenuation.
- *c_ijk_​*: The change in state fatigue from pre‐ to post‐in the *i*‐th group, during task *j*, for the *k*‐th subject, affecting baseline attenuation.
- *d_ijk_* ​: The change in fatigability in the *i*‐th group, during task *j*, for the *k*‐th subject,
- *u*_0_ *_i_*: Random intercept for group *i*, capturing group‐level variability
- ϵ*_ijk_* ​: Residual error term, representing random variability and measurement error not explained by the fixed effects.

Hypothesis 4 Among individuals with long COVID, trait fatigue will be affected by mood, illness beliefs, autonomic symptoms, increase in state fatigue induced by a physical task, the increase in state fatigue induced by a cognitive task, and autonomic nervous system function.

This was tested using a linear mixed effects model:

$$Y_{i}=\mu +a_{i}+b_{i}+c_{i}+d_{i}+e_{i}+f_{i}+u_{0i}+\varepsilon_{i}$$

- *Y_i_* ​: Trait fatigue for individual *i*.
- μ is the overall mean of state fatigue following exertion.
- *a_i_* ​: Mood for individual *i*.
- *b_i_*: Illness beliefs for individual *i*.
- *c_i_​*: Autonomic symptoms for individual *i*.
- *d_i_*: Change in state fatigue induced by physical task for individual *i*.
- *e_i_​*: Change in state fatigue induced by cognitive task for individual *i*.
- *f_i_*: autonomic nervous system function for individual *i*
- *u*_0_ *_i_​*: Random intercept capturing individual‐specific variability in trait fatigue not explained by the fixed effects.
- ϵ*_i_​*: Residual error term representing random variability and measurement error.

​ We checked the following assumptions of the regression models: linearity, independence, homoscedasticity, normality and multicollinearity.

Planned exploratory analysis:

- Hypotheses 1–3 were rerun with age included as a covariate.
- Hypotheses 1–4 were rerun with time of day (morning/afternoon) at which participants completed sessions and session order (cognitive/physical first) included as additional predictor variables.
- The effect of autonomic dysfunction during exertion, on state fatigue pre‐ to post‐exertion, via an effect on somatosensory processing post‐exertion was tested using mediation analysis with separate models for somatosensory attenuation and somatosensory gating. This was quantified in participants with and without long COVID.
- The relations between autonomic dysfunction during exertion, state fatigue during exertion and perceived effort during exertion were quantified in participants with and without long COVID during both tasks (cognitive/physical). This was tested using correlation analysis.
- Test–retest reliability was evaluated for each somatosensory processing measure (gating and attenuation) using the baseline measure from each session (cognitive/physical). This provides a measure of whether somatosensory processing is stable over time.

We checked outcome neutral criteria that must be met for successful testing of the stated hypothesis. This included checking for the absence of floor or ceiling effects. This was done by examining data distributions to ensure that there were no extreme values that could impact the validity of results. Additionally, we investigated the quality of the data, including frequency measures and the shape of the distribution.

### 2.8 Sample size

The study was powered to address Research Question 1a. Previous research indicated an effect size of 0.78 for differences in somatosensory attenuation between young and older individuals (Parthasharathy et al., 2022). Powering the current study to detect an effect size of 0.78 between two independent means for Research Question 1a – somatosensory attenuation in individuals with and without long COVID with an α error probability of 0.05 and a power of 95% and based on a non‐directional two‐tailed hypothesis required 44 participants per group (G*power 3.1.9.7). Dropout was not expected to impact Research Question 1a or 1b, as it is evaluated using measurements made at the start of the first session. However, there was potential for drop out to impact the subsequent research questions which rely on participants returning for a second session.

The target sample size gave us power of 90% to detect an effect of 0.70 between two groups at two repeated time points with α error probability of 0.05 for question 2 and 3, or a power of 80% to detect an effect of 0.60. A dropout rate of 10% would mean that our power to detect these effect sizes would decrease to 60%. The approach for addressing research questions 2 and 3 ensures that the study is adequately powered to detect correlations of 0.50 and 0.40, respectively. The code used to calculate the outcome measure is available: https://github.com/smancuso/R‐Code/blob/cc90c623c4dc733fe5c93ef5a795dde010152c63/Power.R

## 3 RESULTS

A total of 126 participants (55 controls, 71 long COVID) completed the online consent, initial screening and self‐report measures. Of these, 38 participants (11 controls, 27 long COVID) did not continue to the in‐person testing session. The remaining 88 participants attended at least one in‐person testing session (44 controls, 44 long COVID), meeting our a priori sample size calculation. Data from these 88 participants are reported throughout the main text (Table 2). Data from participants that completed the self‐report measures only (*n* = 38) are reported in Supplementary material 7. Data procedures and handling of missing data are detailed in Supplementary material 8.

**TABLE 2:** Characteristics of study sample.

	Control (*n* = 44)	Long COVID (*n* = 44)
Age (years)	30.0 [24.0–50.5]	52.0 [42.5–60.5]
Sex		
Female	26 (59.1)	33 (75.0)
Male	17 (38.6)	10 (22.7)
Other	1 (2.3)	1 (2.3)
Ethnicity		
Asian	6 (13.6)	2 (4.6)
Black	2 (4.5)	1 (2.3)
Mixed or multiple	1 (2.3)	0 (0)
Other	2 (4.5)	0 (0)
Prefer not to say	0 (0)	0 (0)
White	33 (75.0)	41 (93.2)
Test for COVID‐19 infection		
Clinical diagnosis	0 (0)	6 (13.6)
PCR	7 (15.9)	18 (40.9)
LFT	25 (56.8)	17 (38.6)
Other	0 (0)	3 (6.8)
No COVID‐19 infection	12 (27.3)	0 (0)

*Note*: Values are median [interquartile range] or *n* (%). The equivalent information for all participants who completed only the online questionnaires is provided in Supplementary Materials 7, Table 1.

Abbreviations: LFT, Lateral flow test; PCR, Polymerase chain reaction test.

### 3.1 Descriptive statistics across psychometric and clinical measures

**FIGURE 3:**
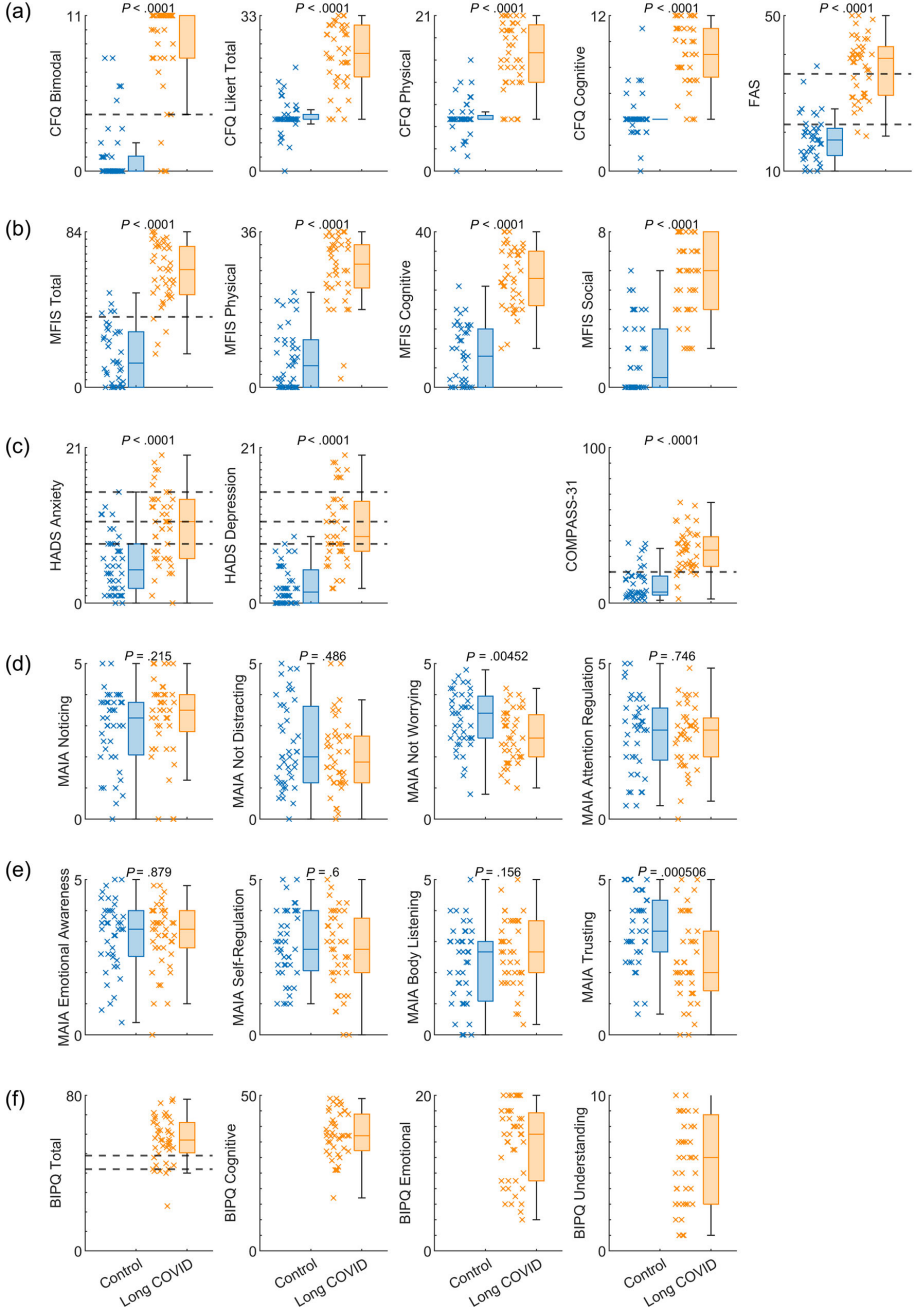
Distribution across measures used to evaluate fatigue severity (a), impact of fatigue on functioning (b), mood (c), autonomic symptoms (c), interoceptive sensibility (d, e) and illness perceptions (f) for control (blue) and long COVID (orange) participants who attended at least one in‐person testing session. Sample sizes vary by measure due to missing data (control *n* = 42–44; long COVID *n* = 43–44; see Table 3 for details). ‘*n*’ represents the number of independent participants contributing to each measure. The *y*‐axes are scaled to show the full range of possible scores on each measure. Boxes show the median and interquartile range; whiskers extend to the most extreme data points within 1.5× the interquartile range. Individual data points are shown by crosses to the left of each box plot. Black dashed lines show clinical severity cutoffs: FAS < 22 = normal, 22–34 = mild‐to‐moderate, ≥35 = severe; CFQ‐11 bimodal ≥4 = excessive fatigue; MFIS ≥38 = high impact; COMPASS‐31 0–20 = mild, 21–100 = moderate‐severe; B‐IPQ < 42 = low, 42–49 = moderate, ≥50 = high threat. The equivalent plot for all participants who completed the online questionnaires is provided in Supplementary material 7, Figure 1. BIPQ, Brief Illness Perception Questionnaire; CFQ‐11, Chalder Fatigue Questionnaire; COMPASS‐31, Composite Autonomic Symptom Score; FAS, Fatigue Assessment Scale; HADS, Hospital Anxiety and Depression Scale; MAIA, Multidimensional Assessment of Interoceptive Awareness; MFIS, Modified Fatigue Impact Scale.

Figure 3 presents the distribution of scores across all study measures for the 88 participants who attended at least one in‐person testing session (44 controls, 44 long COVID), with measures of central tendency (median) and spread (interquartile range) reported in Table 3.

**TABLE 3:** Descriptive statistics of psychometric and clinical measures.

	*n*	Control	*n*	Long COVID	*Z*‐score	*P*
CFQ bimodal	44	0.00 [0.00–1.05]	43	11.00 [8.00–11.00]	−7.255	<0.0001
CFQ Likert	44	11.00 [11.00–12.00]	43	25.00 [20.00–31.00]	−7.248	<0.0001
CFQ physical	44	7.00 [7.00–7.50]	43	16.00 [12.00–19.75]	−6.818	<0.0001
CFQ cognitive	44	4.00 [4.00–4.00]	43	9.00 [7.25–11.00]	−7.132	<0.0001
FAS	44	18.00 [14.00–21.00]	44	39.00 [29.50–42.00]	−7.511	<0.0001
MFIS total	42	13.00 [0.00–30.00]	42	63.50 [50.00–76.00]	−7.385	<0.0001
MFIS physical	42	5.00 [0.00–11.00]	42	28.50 [23.00–33.00]	−7.247	<0.0001
MFIS cognitive	42	8.00 [0.00–15.00]	42	28.00 [21.00–35.00]	−7.348	<0.0001
MFIS social	42	0.50 [0.00–3.00]	42	6.00 [4.00–8.00]	−6.882	<0.0001
HADS total	44	7.00 [2.50–12.00]	43	20.00 [13.25–26.00]	−6.229	<0.0001
HADS – anxiety	44	4.50 [2.00–8.00]	43	11.00 [6.00–14.00]	−4.606	<0.0001
HADS – depression	44	1.50 [0.00–4.50]	43	9.00 [7.00–13.75]	−6.937	<0.0001
BIPQ total	—	—	43	57.00 [50.50–66.00	—	—
BIPQ cognitive	—	—	43	37.00 [32.25–44.00]	—	—
BIPQ emotional	—	—	43	15.00 [9.00–17.75]	—	—
BIPQ understanding	—	—	43	6 [3.00–8.75]	—	—
COMPASS‐31	44	7.14 [5.12–17.32]	44	34.07 [23.67–42.67]	−6.464	<0.0001
COMPASS orthostatic	44	0.00 [0.00–12.00]	44	16.00 [12.00–20.00]	−5.390	<0.0001
COMPASS vasomotor	44	0.00 [0.00–0.00]	43	0.00 [0.00–2.50]	−3.737	0.000186
COMPASS secretomotor	44	0.00 [0.00–2.14]	43	4.29 [2.14–8.04]	−4.993	<0.0001
COMPASS gastrointestinal	44	4.02 [1.79–6.25]	43	7.14 [5.36–10.71]	−4.0428	<0.0001
COMPASS bladder	44	0.00 [0.00–0.00]	44	1.11 [0.00–2.22]	−4.518	<0.0001
COMPASS pupillomotor	44	0.33 [0.00–1.33]	43	2.33 [1.33–3.00]	−5.0949	<0.0001
MAIA noticing	43	3.25 [2.06–3.75]	43	3.5	−1.241	0.215
MAIA not distracting	43	2 [1.17–3.63]	43	1.833	0.696	0.486
MAIA not worrying	43	3.4 [2.6–3.95]	43	2.6	2.840	0.00453
MAIA attention regulation	43	2.86 [1.89–3.57]	43	2.86	0.324	0.746
MAIA emotional awareness	43	3.4 [2.53–4]	43	3.4	.152	.879
MAIA self‐regulation	43	2.75 [2.06–4]	43	2.75	0.524	0.600
MAIA body listening	43	2.67 [1.08–3]	43	2.67	−1.419	0.156
MAIA trusting	42	3.33 [2.67–4.33]	43	2	3.477	0.000506

*Note*: Values are median [interquartile range]. The equivalent information for all participants who completed only the online questionnaires is provided in Supplementary material 7, Table 2.

Abbreviations: BIPQ, Brief Illness Perception Questionnaire; CFQ‐11, Chalder Fatigue Questionnaire; COMPASS‐31, Composite Autonomic Symptom Score; FAS, Fatigue Assessment Scale; HADS, Hospital Anxiety and Depression Scale; MAIA, Multidimensional Assessment of Interoceptive Awareness; MFIS, Modified Fatigue Impact Scale.

Among the long COVID participants, severe fatigue was reported by 64% according to FAS score and 86% according to CFQ‐11 bimodal score, with 91% reporting high impact of fatigue (MFIS score; Figure 3). Autonomic dysfunction was moderate/severe in 89%. Perceived illness threat of long COVID was high in 77%. Severe anxiety was reported by 53% and severe depression by 42%.

There was no significant difference in sex distribution between the long COVID and control groups (χ^2^ (2, *n* = 88) = 2.65, *P *= 0.266). The Shapiro–Wilk test indicated non‐normal distributions for many self‐report scales, and therefore the Mann–Whitney *U*‐test was used to compare these across groups. As shown in Table 3, individuals with long COVID reported substantially greater trait fatigue across all measures (FAS, CFQ‐11 total, physical and cognitive subscales, MFIS total, physical and cognitive subscales) compared with controls. They also reported higher levels of anxiety and depression (HADS‐A, HADS‐D) and more severe autonomic symptoms across all COMPASS‐31 domains. Differences in interoceptive awareness (MAIA) varied across subscales. BIPQ measures could not be compared as the control group could not report on beliefs about having an illness. All group differences were maintained when analyses were repeated including data from the participants who did not attend an in‐person testing session (Supplementary material 7, Table 3).

#### 3.1.1 In‐person testing sessions

Shapiro–Wilk tests indicated that the distribution of variables deviated from normality within groups. Therefore, all descriptive statistics are reported using median and interquartile range as robust measures of central tendency and spread.

**FIGURE 4:**
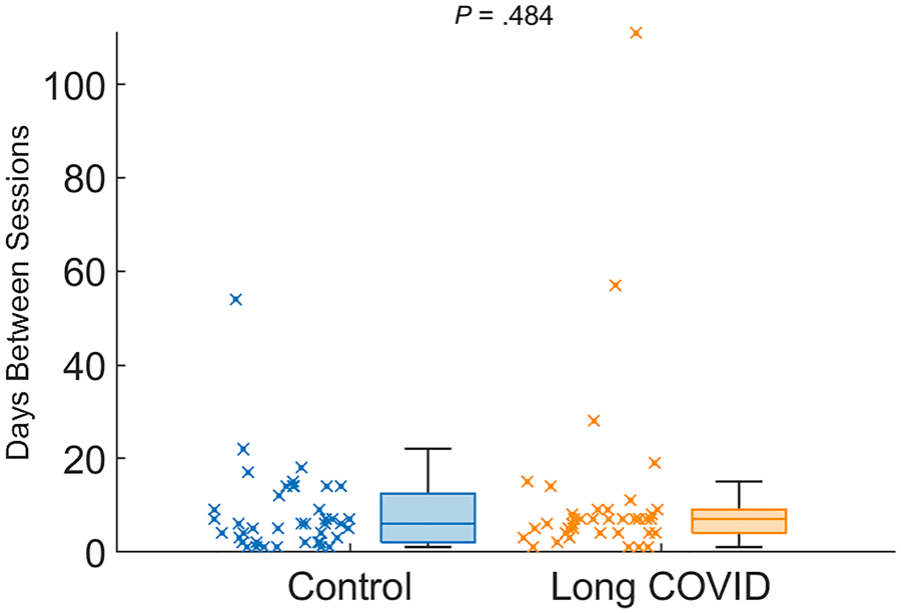
Number of days between consecutive testing sessions for control (blue)and long COVID (orange) participants. Boxes show the median and interquartile range; whiskers extend to the most extreme data points within 1.5× the interquartile range. Individual data points are shown by crosses to the left of each box plot. Sample sizes vary due to some participants only completing one session (long COVID: *n* = 38; control: *n* = 41). ‘*n*’ represents the number of independent participants contributing to each measure.

Session order was randomised: 44 participants (19 control, 25 long COVID) undertook the cognitive session first, and 44 (25 control, 19 long COVID) undertook the physical session first. Nine participants (three controls, six long COVID) did not return for their second session; therefore, 167 sessions were held in total. Of the missed second sessions, four were cognitive (two long COVID, two control) and five were physical (four long COVID, one control). This resulted in 84 participants completing the cognitive session (42 long COVID; 42 control) and 83 participants completing the physical session (*n* = 40 long COVID; *n* = 43 control). Two sessions (one cognitive, one physical) were stopped early at the participant's request, both were individuals with long COVID. For further details see Supplementary material 8. Data collected up to the point of stopping are included in the analysis. No adverse events were reported following the sessions. The median interval between the first and second session was 7 days (Figure 4). All sessions occurred between 09.00 and 17.30 h.

**FIGURE 5:**
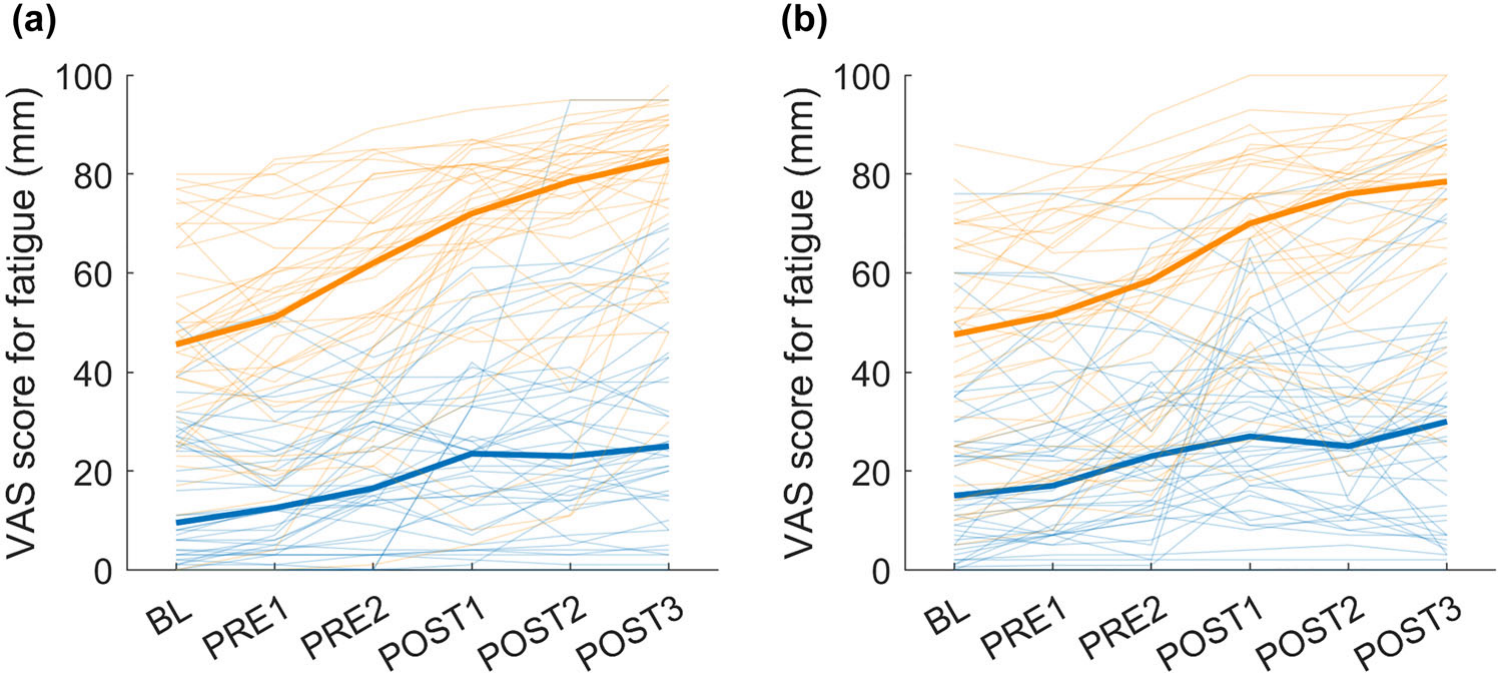
Visual analogue scale (VAS) score for fatigue at each of six time points throughout the cognitive (VAS; a) and physical (VAS; b) testing session for control (blue) and long COVID (orange) participants. Thin lines represent individual data points. Thick lines represent median values. The VAS scale was 100 mm long and anchored on the left side (0 mm) with ‘not fatigued at all’ and on the right side (100 mm) with ‘extremely fatigued’. VAS score for fatigue was evaluated at baseline (BL), immediately after pre‐task somatosensory attenuation measure (PRE‐1), immediately after pre‐task somatosensory gating measure (PRE‐2), immediately after the completion of the task (cognitive or physical) (POST‐1), immediately after post‐task somatosensory attenuation measure (POST‐2) and immediately after post‐task somatosensory gating measure (POST‐3). Sample sizes were consistent across time points for controls (cognitive: *n* = 42; physical: *n* = 43) but varied slightly for long COVID participants (cognitive: *n* = 41–42; physical: *n* = 38–40) due to missing data and incomplete sessions. ‘*n*’ represents the number of independent participants contributing to each measure.

At the start of the testing session (BL), individuals with long COVID reported greater state fatigue than controls in the cognitive session (*n* = 42 long COVID, *n* = 42 controls; VAS score: 45.5 [32.0–65.0] mm for long COVID vs. 9.5 [3.0–27.0] mm for controls) and the physical session (*n* = 40 long COVID, *n* = 43 controls; 47.5 [25.0–65.0] mm vs. 15.0 [5.3–29.0] mm; Figure 5). This group difference in state fatigue was maintained throughout the testing session (Mann–Whitney *U*‐test uncorrected *P* < .001 at all time points; Figure 5).

**FIGURE 6:**
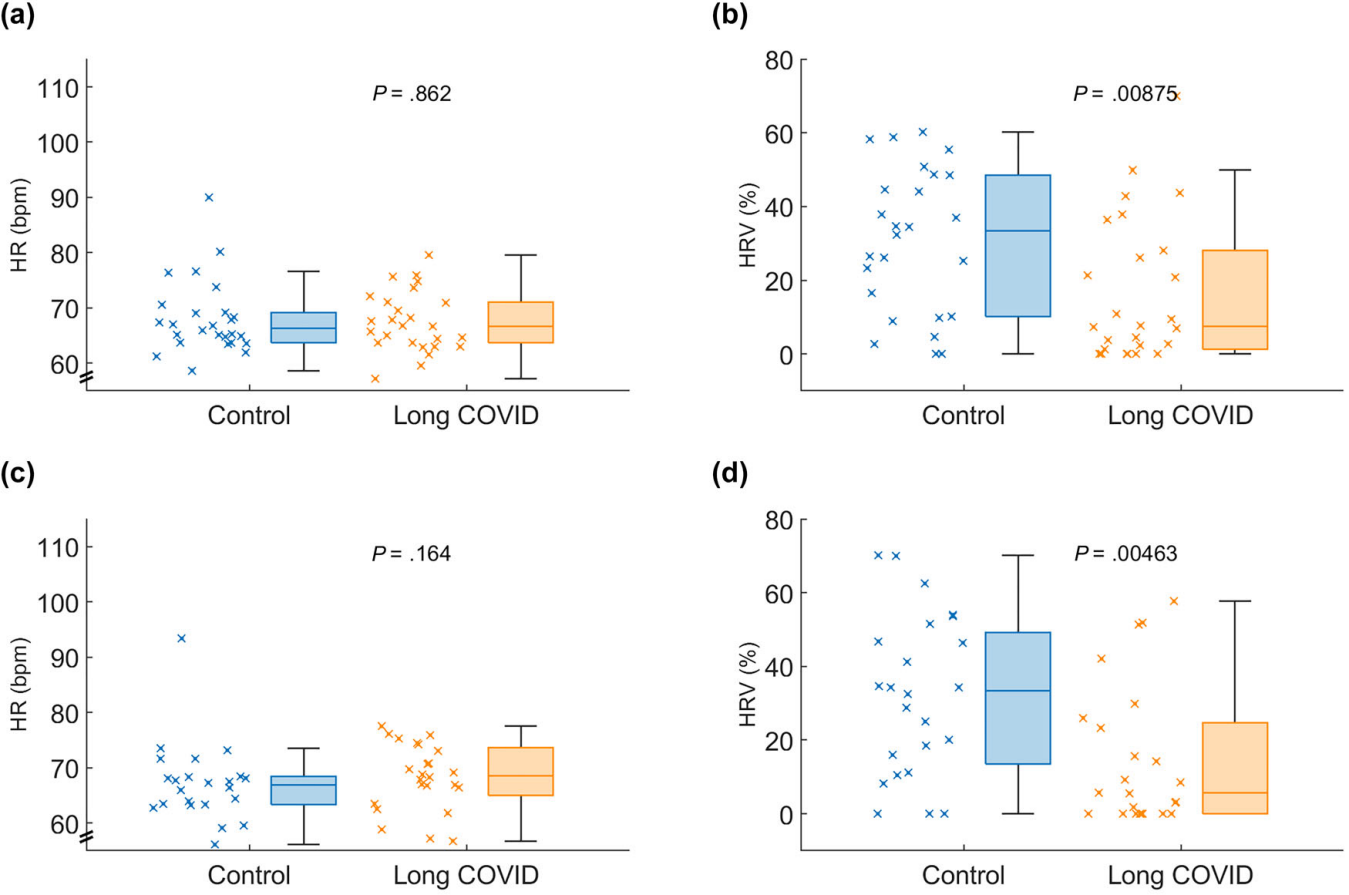
Heart rate and heart rate variability measured during quiet sitting in the pre‐task assessment of sensory attenuation. Heart rate (HR; a, c) and heart rate variability (HRV; b, d) measured during quiet sitting in the pre‐task assessment of sensory attenuation in the cognitive (a, b) and physical (c, d) testing sessions for control (blue) and long COVID (orange) participants. Boxes show the median and interquartile range; whiskers extend to the most extreme data points within 1.5× the interquartile range. Individual data points are shown by crosses to the left of each box plot. HRV was calculated as percentage of successive inter‐beat intervals that differed by more than 50 ms. Sample sizes vary across the cognitive (long COVID: *n* = 26; control: *n* = 26) and physical sessions (long COVID: *n* = 24; control: *n* = 24) due to missing data and incomplete sessions. ‘*n*’ represents the number of independent participants contributing to each measure. Mann–Whitney *U*‐test *P*‐values for group comparisons are displayed within the figure.

Heart rate recordings that were of poor quality, defined as those with excessive noise or artefacts precluding reliable identification of heart beats upon visual inspection, were excluded from analysis. Data were processed for the remaining participants only (26 long COVID and 26 controls in the cognitive session and 24 long COVID and 24 controls in the physical session). At the start of the testing session (during pre‐task sensory attenuation measures), individuals with long COVID had similar heart rate (HR) as controls (73.4 [67.3–82.0] bpm vs. 72.6 [67.5–78.4] bpm in the cognitive session and 77.0 [70.0–87.3] bpm vs. 73.6 [66.6–76.7] bpm in the physical session; Mann–Whitney *U*‐test uncorrected *P* = 0.862 and 0.164, respectively; Figure 6). However, individuals with long COVID had lower HRV than controls (7.5 [1.3–28.1]% vs. 33.4 [10.1–48.5]% in the cognitive session and 5.7 [0.0–24.7]% vs. 33.4 [13.5–49.2]% in the physical session, Mann–Whitney *U*‐test uncorrected *P* = 0.00875 and 0.00463).

**FIGURE 7:**
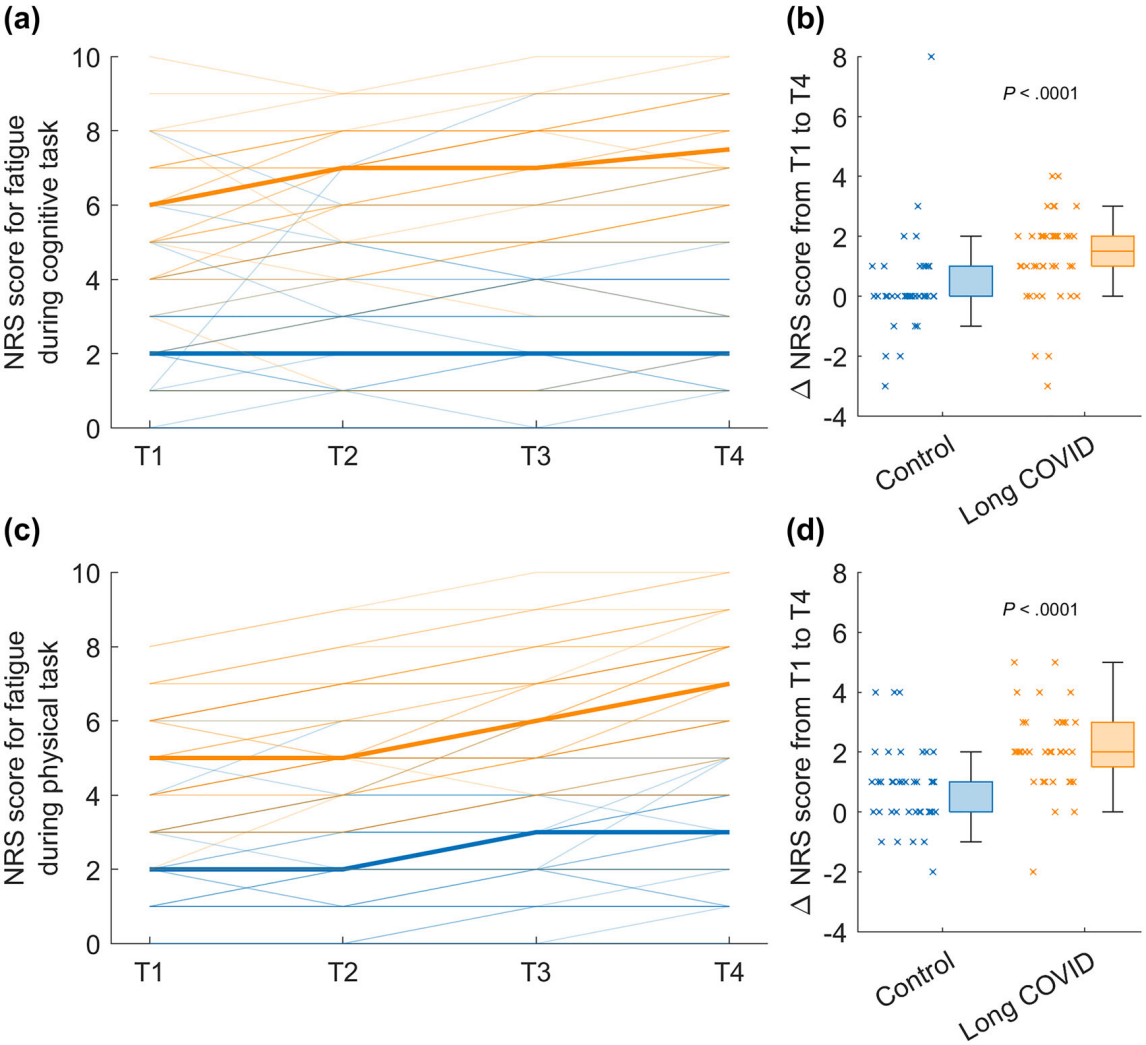
Fatigue during cognitive (top row) and physical (bottom row) tasks. Left panels (a, c) show numerical rating scale (NRS) score at each of four time points during the cognitive task (a) and the physical task (c) for control (blue) and long COVID (orange) participants. Right panels (b, d) show change in NRS score from the first to the fourth time point during the cognitive task (b) and the physical task (d). Boxes show the median and interquartile range; whiskers extend to the most extreme data points within 1.5× the interquartile range. Individual data points are shown by crosses to the left of each box plot. Time points (T1–T4) indicate when NRS ratings were collected: after every fourth task and at the end of the cognitive session, and at 90 s, 180 s, 270 s and at the end of the 6‐min walk test for the physical task. Sample sizes vary across the cognitive (long COVID: *n* = 42; control: *n* = 42) and physical sessions (long COVID: *n* = 40; control: *n* = 43) due to missing data and incomplete sessions. ‘*n*’ represents the number of independent participants contributing to each measure. Mann–Whitney *U*‐test *P*‐values for group comparisons are displayed within the figure.

**FIGURE 8:**
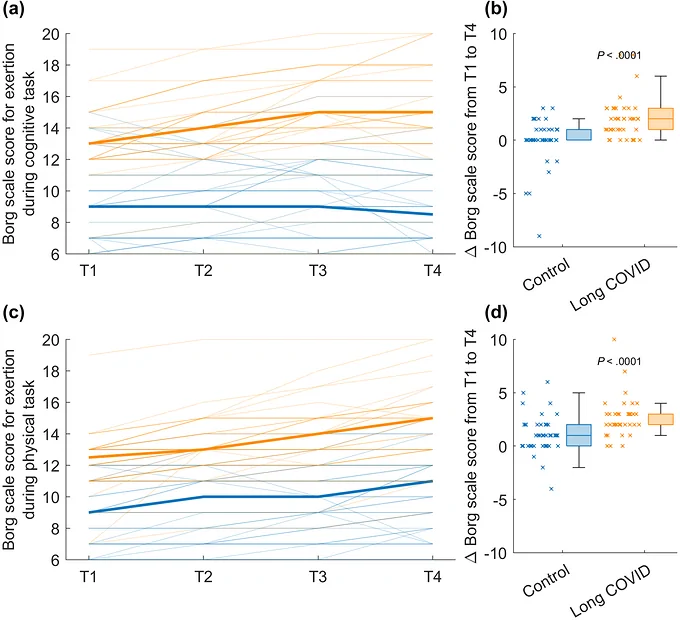
Perceived exertion during cognitive (a, b) and physical (c, d) tasks. Left panels (a, c) show Borg rating of perceived exertion score at each of four time points during the cognitive task (a) and the physical task (c) for control (blue) and long COVID (orange) participants. Right panels (b, d) show change in Borg score from the first to the fourth time point during the cognitive task (b) and the physical task (d). Boxes show the median and interquartile range; whiskers extend to the most extreme data points within 1.5× the interquartile range. Individual data points are shown by crosses to the left of each box plot. Time points (T1–T4) indicate when Borg ratings were collected: after every fourth task and at the end of the cognitive session, and at 90 s, 180 s, 270 s and at the end of the 6‐min walk test for the physical task. Sample sizes vary across the cognitive (long COVID: *n* = 42; control: *n* = 42) and physical sessions (long COVID: *n* = 40; control: *n* = 43) due to missing data and incomplete sessions. ‘*n*’ represents the number of independent participants contributing to each measure. Mann–Whitney *U*‐test *P*‐values for group comparisons are displayed within the figure.

During both the cognitive and the physical tasks, long COVID participants reported greater state fatigue (NRS score; Figure 7a) and perceived exertion (RPE scale; Figure 8a) than controls at all time points (Mann–Whitney *U*‐test uncorrected *P* < 0.0001 at all time points in both sessions for both variables; cognitive session: *n* = 42 long COVID, *n* = 42 controls; physical session: *n* = 40 long COVID, *n* = 43 controls). In the cognitive task, state fatigue and perceived exertion increased from the first to the fourth (final) time point for the long COVID group (both Wilcoxon signed‐rank test uncorrected *P* < 0.0001) but did not change in the control group (*P* = 0.282 and 0.706, respectively; Figures 7b and 8b, respectively). In the physical task, state fatigue and perceived exertion increased from the first to the final time point in both groups (*P* ≤ 0.0001 and 0.000457, respectively; Figure 7d and 8d, respectively). In both the cognitive and the physical task, the change in state fatigue and perceived exertion from the first to the final time point was greater in the long COVID group than in the control group (all Mann–Whitney *U*‐test uncorrected *P* < 0.0001; Figures 7b,d and 8b,d).

**FIGURE 9:**
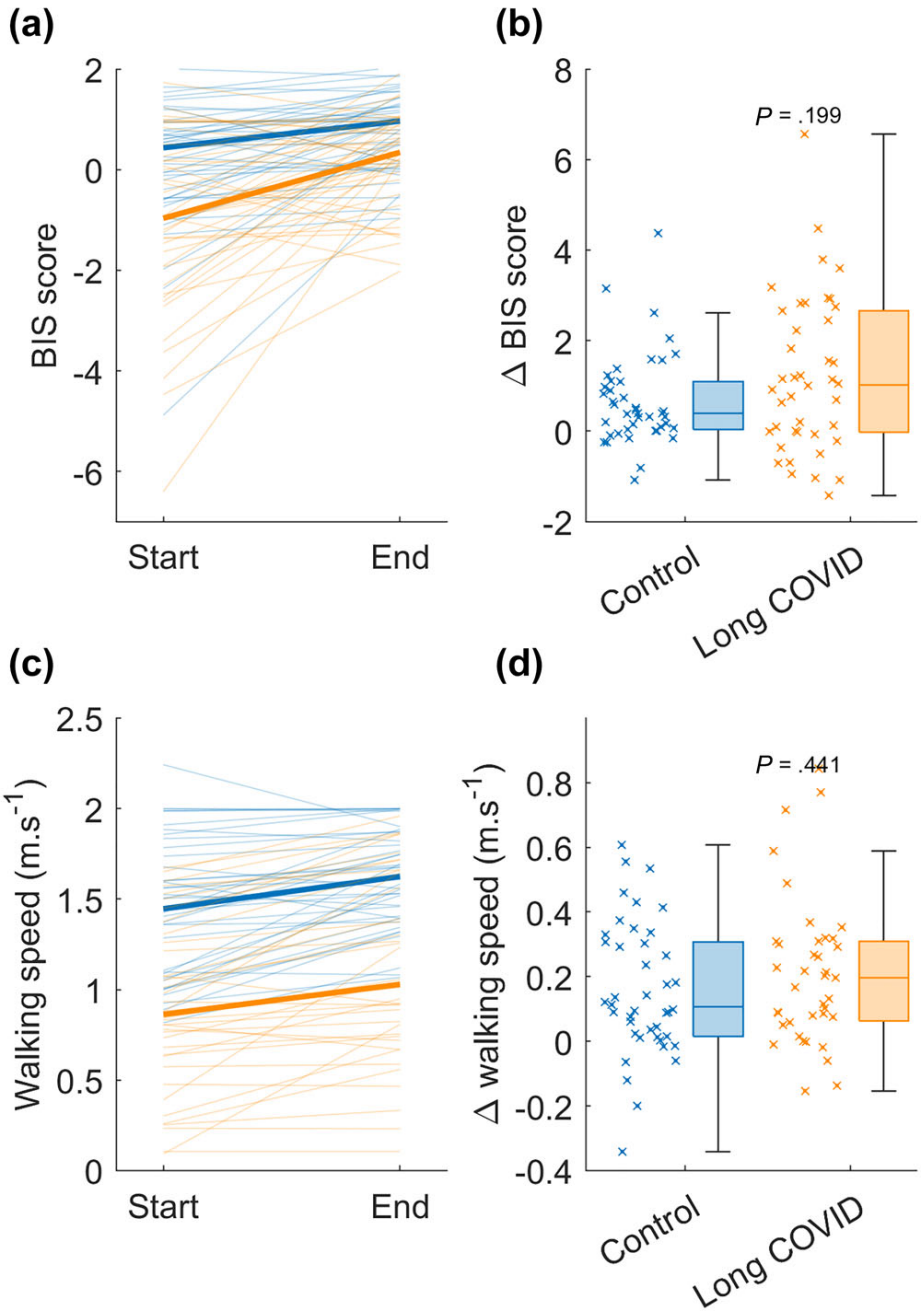
Task performance during the cognitive (a, b) and physical (c, d) tasks. Left panels (a, c) show performance at the start and end of the cognitive task (a) and the physical task (c) for control (blue) and long COVID (orange) participants. Right panels (b, d) show change in performance (fatigability) across the task during the cognitive task (ΔBIS) (b) and the physical task (Δ walking speed) (d). Boxes show the median and interquartile range; whiskers extend to the most extreme data points within 1.5× the interquartile range. Individual data points are shown by crosses to the left of each box plot. For the cognitive task, performance was evaluated using the balanced integration score (BIS) in the AX‐CPT task at the start and end of the cognitive testing battery. For the physical task, performance was evaluated using average walking speed at the start (30–60 s) and end (330–360 s) of the 6‐min walk test. Sample sizes were consistent across time points for the cognitive task (long COVID: *n* = 42; controls: *n* = 42) but varied slightly for the physical session (long COVID: *n* = 37–39; control: *n* = 36–42) due to missing data. ‘*n*’ represents the number of independent participants contributing to each measure.

During the cognitive task, the BIS score was lower in long COVID participants (*n* = 42) than controls (*n* = 42) at both the first (−0.96 [−1.93–0.18] vs. 0.44 [−0.97–0.94]) and the final (0.35 [−0.49–0.91] vs. 0.97 [0.13–1.32]) time point (Mann–Whitney *U*‐test uncorrected *P* = 0.000686 and *P* = 0.00359, respectively). During the physical task the total distance walked was less in long COVID participants (*n* = 39; 333.7 [256.4–477.2] m) than controls (*n* = 42; 561.5 [468.4–606.1] m) (Mann–Whitney *U*‐test uncorrected *P* < 0.0001). During both the cognitive and the physical tasks, long COVID participants had a similar change in performance (fatigability) from the start to the end of the task as controls. Cognitive fatigability (ΔBIS) was 1.03 [−0.02–2.67] in the long COVID group (*n* = 42) and 0.39 [0.04–1.09] in the control group (*n* = 42) (*P* = 0.199; Figure 9b). Physical fatigability (Δ walking speed) was 0.17 [0.06–0.31] m s^−1^ in the long COVID group (*n* = 37) and 0.13 [0.04–0.33] m s^−1^ in the control group (*n* = 36; *P* = 0.441; Figure 9d). In both the cognitive and the physical tasks, performance increased from the start to the end in both groups (both Wilcoxon signed‐rank test uncorrected *P* < 0.0001; Figure 9a,c, respectively).

Heart rate recordings that were of poor quality were excluded from analysis. To maximise the number of participants retained in HR analyses, only data from the first and the final minute of the task were analysed. HR and HRV during the tasks were calculated for the included participants only (*n* = 20 long COVID and 20 controls in the cognitive task and 12 long COVID and 12 controls participants in the physical task).

**FIGURE 10:**
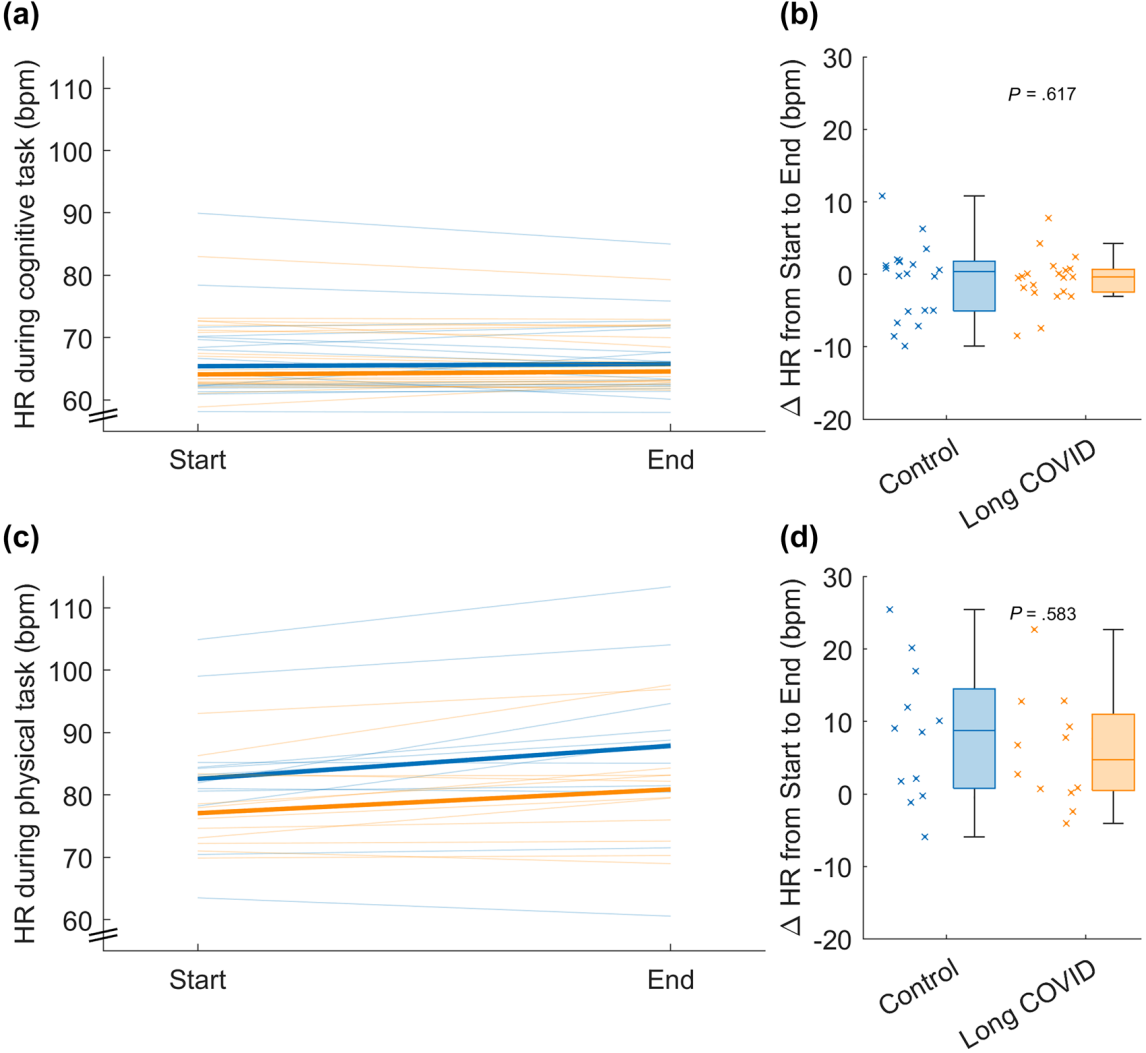
Heart rate (HR) during cognitive (a, b) and physical (c, d) tasks. Left panels (a, c) show HR at each of four time points during the cognitive task (a) and the physical task (c) for control (blue) and long COVID (orange) participants. Right panels (b, d) show change in HR from the first to the fourth time point during the cognitive task (b) and the physical task (d). Boxes show the median and interquartile range; whiskers extend to the most extreme data points within 1.5× the interquartile range. Individual data points are shown by crosses to the left of each box plot. Sample sizes vary across the cognitive (long COVID: *n* = 20; control: *n* = 20) and physical sessions (long COVID: *n* = 12; control: *n* = 12) due to missing data and incomplete sessions. ‘*n*’ represents the number of independent participants contributing to each measure. Mann–Whitney *U*‐test *P*‐values for group comparisons are displayed within the figure.

In the cognitive task, HR was similar in long COVID participants compared to controls at both the start (66.8 [65.5–82.8] bpm vs. 70.7 [64.2–80.0] bpm) and the end (70.9 [65.7–80.0] bpm vs. 71.2 [64.7–77.3 bpm]) time point (Mann–Whitney *U*‐test uncorrected *P* = 0.452 and *P* = 0.688, respectively). In the physical task HR was similar in long COVID participants and controls at both the start (95.9 [85.1–106.6] bpm vs. 103.9 [101.4–110.0] bpm and the end (104.3 [90.3–109.4] bpm vs. 115.7 [101.9–125.1] bpm) time point (Mann–Whitney *U*‐test uncorrected *P* = 0.135 and *P* = 0.149, respectively). In the cognitive task, HR did not change from the start to the end in either the long COVID or the control group (Wilcoxon signed‐rank test uncorrected, *P* = 0.279 and *P* = 0.737, respectively; Figure 10a). In the physical task, HR increased from the start to the end in both groups (*P* = 0.0229 and 0.01502, respectively; Figure 10c). During both the cognitive and the physical tasks, long COVID participants had a similar change in HR from the start to the end of the task as controls (Mann–Whitney *U*‐test uncorrected *P* = 0.617 and 0.583, respectively; Figure 10b,d).

**FIGURE 11:**
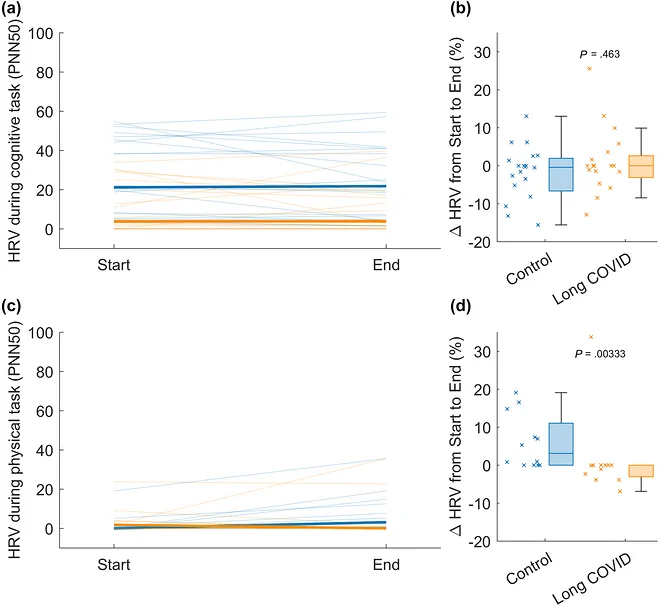
Heart rate variability (HRV) during cognitive (a, b) and physical (c, d) tasks. Left panels (a, c) show HRV at each of four time points during the cognitive task (a) and the physical task (c) for control (blue) and long COVID (orange) participants. Right panels (b, d) show change in HRV from the first to the fourth time point during the cognitive task (b) and the physical task (d). Boxes show the median and interquartile range; whiskers extend to the most extreme data points within 1.5× the interquartile range. Individual data points are shown by crosses to the left of each box plot. Sample sizes vary across the cognitive (long COVID: *n* = 20; control: *n* = 20) and physical sessions (long COVID: *n* = 12; control: *n* = 12) due to missing data and incomplete sessions. ‘*n*’ represents the number of independent participants contributing to each measure. Mann–Whitney *U*‐test *P*‐values for group comparisons are displayed within the figure.

In the cognitive task, the HRV was different in long COVID participants than controls at both the start (4.4 [0–21.5]% vs. 33.9 [7.9–48.6]%) and the end (4.4 [1.4–17.1]% vs. 19.8 [4.7–41.0]%) time point (Mann–Whitney *U*‐test uncorrected *P *= 0.000979 and *P* = 0.014003, respectively). In the physical task HRV was similar in long COVID participants and controls at both the start (0 [0–3.5]% vs. 0 [0–2.7]% and the end (0 [0–1.7]% vs. 3.1 [0–13.7]%) time point (Mann–Whitney *U*‐test uncorrected *P* = 0.748 and *P* = 0.151, respectively). In the cognitive task, HRV did not change from the start to the end in either the long COVID or control group (Wilcoxon signed‐rank test uncorrected, *P *= 0.865 and 0.210, respectively; Figure 11a). Long COVID participants had a similar change in HRV from the start to the end of the task as controls (Mann–Whitney *U*‐test uncorrected *P* = 0.463; Figure 11b). In the physical task, HRV did not change from the start to the end in the long COVID group but increased for controls (Wilcoxon signed‐rank test uncorrected, *P *= 0.237 and *P* = 0.0117, respectively; Figure 11c). Long COVID participants had less of an increase in HRV from the start to the end of the task than controls (Mann–Whitney *U*‐test uncorrected *P* = 0.00333; Figure 11d).

**FIGURE 12:**
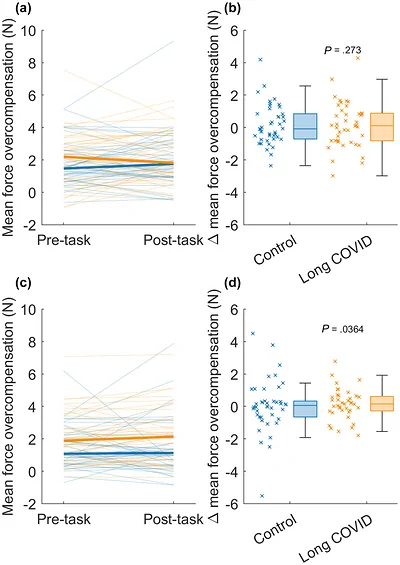
Somatosensory attenuation during the cognitive (a, b) and physical (c, d) testing sessions. Left panels (a, c) show somatosensory attenuation before and after the cognitive task (a) and the physical task (c) for control (blue) and long COVID (orange) participants. Right panels (b, d) show change in somatosensory attenuation from pre‐ to post‐cognitive (b) and physical (d) task. Boxes show the median and interquartile range; whiskers extend to the most extreme data points within 1.5× the interquartile range. Individual data points are shown by crosses to the left of each box plot. Sample sizes vary across both the cognitive (long COVID: *n* = 42; control: *n* = 42) and the physical sessions (long COVID: *n* = 39; control: *n* = 42) due to missing data and incomplete sessions. ‘*n*’ represents the number of independent participants contributing to each measure. Mann–Whitney *U*‐test *P*‐values for group comparisons are displayed within the figure.

Attenuation is reported as mean force overcompensation over all four force levels. At baseline (pre‐task), attenuation was similar in long COVID participants and controls in the cognitive session (2.19 [0.82–2.99] N vs. 1.47 [0.84–2.36] N, respectively, *P* = 0.273; Figure 12a) but was different between groups in the physical session (1.94 [0.95–3.04] N vs. 1.08 [0.50–2.18] N, respectively, *P* = 0.0364; Figure 12c). Although this difference reached conventional statistical significance (0.05), the effect size was small, and the *P*‐value is unadjusted for multiple comparisons; therefore, this result should be interpreted with caution.

**FIGURE 13:**
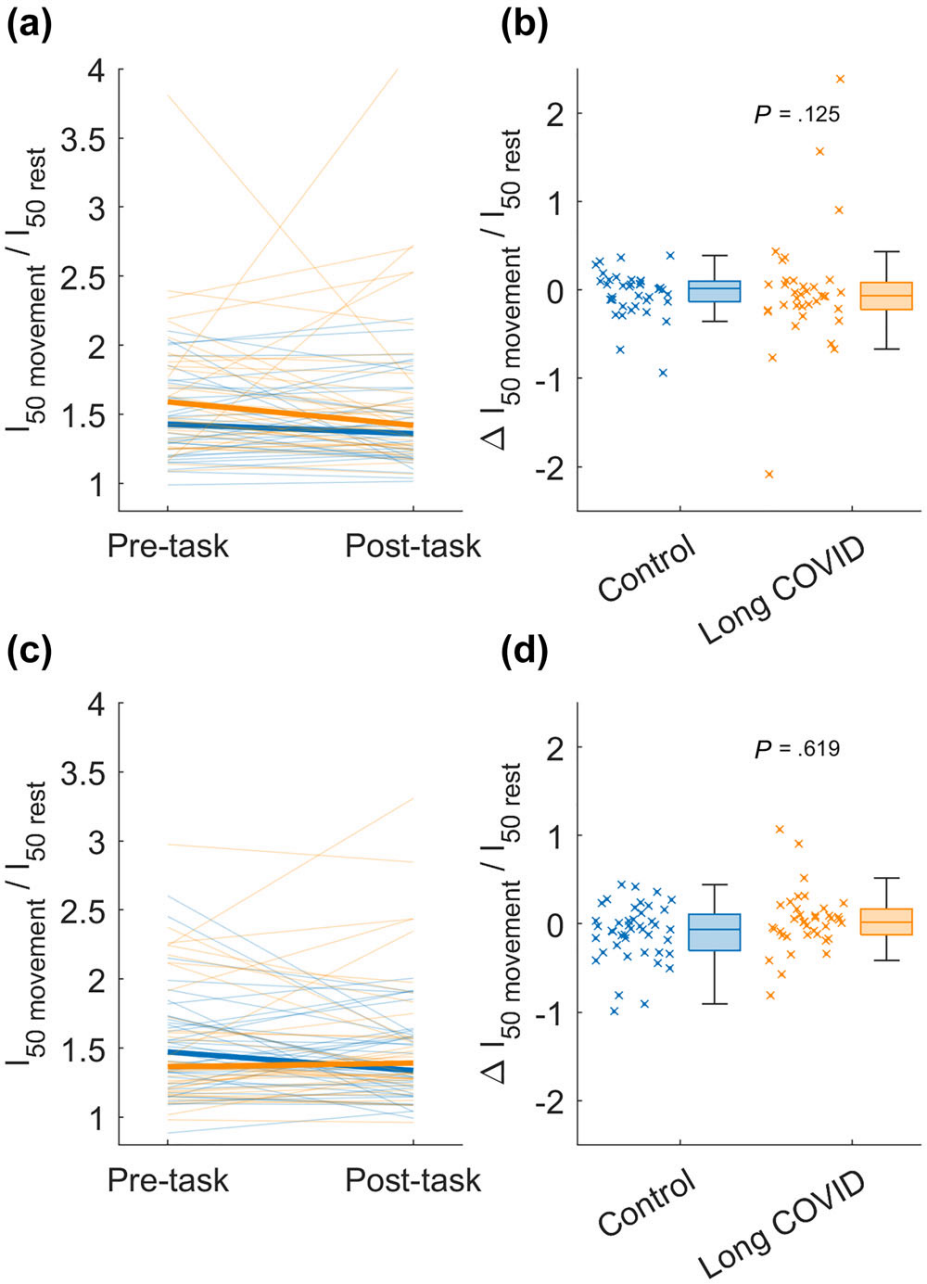
Somatosensory gating during the cognitive (a, b) and physical (c, d) testing sessions. Left panels (a, c) show somatosensory gating before and after the cognitive task (a) and the physical task (c) for control (blue) and long COVID (orange) participants. Right panels (b, d) show change in somatosensory gating from pre‐ to post‐cognitive task (b) and the physical task (d). Boxes show the median and interquartile range; whiskers extend to the most extreme data points within 1.5× the interquartile range. Individual data points are shown by crosses to the left of each box plot. Sample sizes vary across both the cognitive (long COVID: *n* = 36; control: *n* = 38) and the physical sessions (long COVID: *n* = 38; control: *n* = 43) due to missing data and incomplete sessions. ‘*n*’ represents the number of independent participants contributing to each measure. Mann–Whitney *U*‐test *P*‐values for group comparisons are displayed within the figure.

Gating is reported as (*I*_50_rest_/*I*_50_movement_). At baseline (pre‐task), gating was similar in long COVID participants and controls in the cognitive session (1.51 [1.32–1.83] vs. 1.43 [1.28–1.68], respectively, *P *= 0.125; Figure 13a) and in the physical session (1.36 [1.18–1.74] vs. 1.47 [1.26–1.68], respectively, *P *= 0.619; Figure 13c).

Hypothesis 1 Pre‐task (baseline) somatosensory processing will be different between individuals with long COVID fatigue and controls.

This hypothesis was not supported. A MANOVA was conducted to examine differences between controls (*n* = 44) and long COVID participants (*n* = 44) on attenuation and gating measured at the start of the first session. Four multivariate outliers were identified using Mahalanobis distance. Inspection confirmed plausible values, and all cases were retained. Box's *M*‐test indicated a violation of the homogeneity. MANOVA is considered robust against departures from this assumption, and the planned analysis was still conducted. There was no significant multivariate effect of group (Pillai's trace = 0.022, *F*(1,86) = 1.89, *P *= 0.172) on attenuation and gating.

In planned additional analysis, age was not a significant predictor when added as a covariate (Pillai's trace = 0.000, *F*(1, 85) = 0.002, *P *= 0.969) and the main effects remained unchanged. First session type (cognitive, physical; Pillai's trace = 0.003, *F*(1, 85) = 0.255, *P *= 0.615) and time of day (morning, afternoon; Pillai's trace = 0.001, *F*(1, 84) = 0.078, *P *= 0.7801) were not significant predictors.

To ensure robustness, repeating the MANOVA after removing the outliers confirmed no significant findings (*P* = 0.362). As some variables showed non‐normality, follow‐up Mann–Whitney *U*‐tests were conducted. Again, no significant differences were found (attenuation: *P* = 0.061; gating: *P* = 0.215).

Hypothesis 2 State fatigue following exertion will be predicted by somatosensory attenuation at baseline, somatosensory gating at baseline, the presence of long COVID and the type of task.

Residual plots supported linearity and homoscedasticity. Residuals were approximately normally distributed by visual inspection of the histogram and Q–Q plot and multicollinearity was low VIF (Variance Inflation Factor) < 5. Post‐exertion state fatigue was not predicted by somatosensory attenuation at baseline and somatosensory gating at baseline. Post‐exertion state fatigue was predicted by the presence of long COVID (long COVID/controls) but not the type of task (cognitive/physical). Performance fatigability was not a moderator variable in these relations. State fatigue prior to exertion was a significant covariate in this analysis. Analyses included 44 controls and 44 long COVID participants. As the model incorporated session‐level data, participants contributed observations from one or both sessions depending on data availability (Table 4).

**TABLE 4:** Results of linear mixed model predicting post‐exertion state fatigue.

Predictor	Estimate (*b*)	SE	*t*(147)	*P*	95% CI lower	95% CI upper
Intercept	5.915	4.205	1.407	0.162	−2.394	14.225
Group (long COVID)	16.581	2.829	5.862	<0.0001	10.99	22.171
Task type (physical)	2.185	2.311	0.945	0.346	−2.383	6.752
Attenuation	0.470	0.679	0.693	0.489	−0.871	1.812
Gating	1.039	2.403	0.433	0.666	−3.709	5.788
Fatigability	2.225	1.730	1.286	0.20043	−1.194	5.644
State fatigue pre	0.741	0.0517	14.312	<0.0001	0.638	0.842
Group × Fatigability	−2.805	1.919	−1.461	0.146	−6.598	0.988
Task x Fatigability	−2.219	6.158	−0.360	0.719	−14.39	9.952

In a planned additional analysis, when age was added as a covariate, older age was significantly associated with lower post‐task fatigue (*b* = −0.231, SE = 0.069, *t*(146) = −3.333, *P* = 0.0001), and the main effects and interactions remained unchanged (Supplementary material 7, Table 4). When first session type (cognitive, physical; *b* = −0.971, SE = 0.1.982, *t*(145) = −0.490, *P *= 0.625) and time of day (morning, afternoon; *b* = 0.805, SE = 1.93, *t*(145) = 0.417, *P *= 0.678) were added, they were not significant predictors.

Hypothesis 3 The change in somatosensory attenuation (Research Question 3a) and somatosensory gating (Research Question 3b) from pre‐ to post‐task will be predicted by the change in state fatigue from pre‐ to post, fatigability, the presence of long COVID and the type of task.

Residual plots supported linearity and homoscedasticity. Residuals were approximately normally distributed by visual inspection of the histogram and Q–Q plot and multicollinearity was low (VIF < 5).

**Somatosensory attenuation**: The linear mixed‐effects model revealed no significant predictors of change in attenuation from pre‐ to post‐task (Table 5). In a planned additional analysis, when age was added as a covariate, it was not a significant predictor, and the main effects and interactions remained unchanged (Table 6). When first session type (cognitive, physical; *b* = –0.042, SE = 0.200, *P *= 0.835) and time of day (morning, afternoon; *b* = 0.182, SE = 0.198, *P *= 0.3601) were added, they were not significant predictors. This analysis was run with the other measures of attenuation (intercept, slope), and these are presented in Supplementary material 7, Table 5 and 6. Analyses included 44 controls and 44 long COVID participants. As the model incorporated session‐level data, participants contributed observations from one or both sessions depending on data availability.

**TABLE 5:** Results of linear mixed‐effects model predicting change in somatosensory attenuation.

Predictor	Estimate (*b*)	SE	*t*(150)	*P*	95% CI lower	95% CI upper
Intercept	0.256	0.186	1.380	0.1698	−0.111	0.622
Group (long COVID)	0.005	0.206	0.027	0.979	−0.402	0.413
Task (physical)	−0.053	0.210	−0.250	0.80303	−0.468	0.363
Change in fatigue	−0.003	0.008	−0.400	0.689	−0.019	0.013
Performance fatigability	−0.082	0.094	−0.871	0.385	−0.268	0.104

**Somatosensory gating**: The linear mixed effects model revealed no significant predictors of change in gating from pre‐ to post‐task (Table 7). In a planned additional analysis, when age was added as a covariate, it was not a significant predictor, and the main effects and interactions remained unchanged (Table 6). When first session type (cognitive, physical; *b* = –0.045, SE = 0.070, *P *= 0.520) and time of day (morning, afternoon; *b* = 0.030, SE = 0.071, *P *= 0.678) were added, they were not significant predictors. Analyses included 44 controls and 44 long COVID participants. As the model incorporated session‐level data, participants contributed observations from one or both sessions depending on data availability.

**TABLE 6:** Results of linear mixed‐effects model predicting change in somatosensory attenuation and gating including age as a covariate.

	Attenuation				Gating			
Predictor	Estimate (*b*)	SE	*t*(140)	*P*	Estimate (*b*)	SE	*t*(140)	*P*
Intercept	0.644	0.348	1.849	0.066	−1.327	0.114	−11.616	<0.0001
Group (long COVID)	0.181	0.245	0.740	0.461	−0.049	0.082	−0.600	0.549
Task (physical)	−0.057	0.209	−0.274	0.785	0.0573	0.0553	1.037	0.3017
Change in fatigue	−0.006	0.008	−0.696	0.488	−0.00248	0.00246	−1.008	0.315
Fatigability	−0.090	0.094	−0.957	0.340	0.00898	0.0274	0.328	0.743
Age	−0.010	0.008	−1.313	0.191	−0.000898	0.00259	−0.346	0.729

**TABLE 7:** Results of linear mixed‐effects model predicting change in somatosensory gating.

Predictor	Estimate (*b*)	SE	*t*(140)	*P*	95% CI lower	95% CI upper
Intercept	−1.360	0.059	−23.129	<0.0001	−1.477	−1.244
Group (long COVID)	−0.064	0.071	−0.912	0.363	−0.204	0.075
Task (physical)	0.0575	0.0552	1.041	0.299	−0.0517	0.166
Change in fatigue	−0.00233	0.00242	−0.961	0.338	−0.007	0.002
Fatigability	0.00936	0.0274	0.342	0.733	−0.0447	0.0635

Hypothesis 4 Among individuals with long COVID, trait fatigue will be affected by mood, illness beliefs, autonomic symptoms, increase in state fatigue induced by a physical task, the increase in state fatigue induced by a cognitive task and autonomic nervous system function.

Residual plots supported linearity and homoscedasticity. Residuals were approximately normally distributed by visual inspection of the histogram and Q–Q plot and multicollinearity was low (VIF < 5). A linear mixed‐effects model revealed that greater depression (HADS‐D) and perceived illness threat (BIPQ) was associated with greater trait fatigue (FAS) (Table 8). No other variables were significantly associated with trait fatigue.

**TABLE 8:** Results of linear mixed‐effects model predicting trait fatigue in long COVID individuals.

Predictor	Estimate (*b*)	SE	*t*(17)	*P*	95% CI lower	95% CI upper
Intercept	14.558	3.776	3.856	0.00127	6.592	22.524
Anxiety	−0.007	0.228	−0.032	0.975	−0.488	0.474
Depression	1.048	0.211	4.961	<0.0001	0.602	1.494
Illness beliefs	0.286	0.124	2.314	0.0334	0.025	0.547
Autonomic symptoms	0.045	0.076	0.601	0.556	−0.114	0.205
Autonomic function	−0.047	0.037	−1.273	0.2202	−0.125	0.031

**Planned exploratory analysis 1**: The effect of autonomic dysfunction during exertion on state fatigue pre to post exertion, via an effect on somatosensory processing post exertion in participants with and without long COVID.

This was explored using mediation analysis with separate models for somatosensory attenuation and somatosensory gating. During the cognitive session, autonomic function did not predict the change in state fatigue indirectly via attenuation or gating in either group. In long COVID participants, there were no significant predictors of fatigue or mediators. In control participants, autonomic function directly predicted fatigue in the attenuation (*P* = 0.0337) and gating model (*P* = 0.0484) (Supplementary material 7, Table 7 and 8).

During the physical session, autonomic function did not predict the change in state fatigue indirectly via attenuation or gating in either group. In long COVID participants, autonomic function directly predicted attenuation (*P* = 0.00723). In control participants, autonomic function directly predicted gating (*P* = 0.0323), but these mediators did not significantly predict fatigue, and the indirect effects were not significant in any group (Supplementary material 7, Table 9 and 10).

**Planned exploratory analysis 2**: The relationship between autonomic dysfunction during exertion, state fatigue during exertion, and perceived effort during exertion in participants with and without long COVID during both tasks (cognitive/physical).

Exploratory correlation analyses were conducted to examine the relationships between autonomic function (HRV in the final minute of the task), state fatigue (NRS score at the end of the task) and perceived effort (RPE at the end of the task) during both cognitive and physical tasks in participants with and without long COVID. All correlations were calculated using Spearman's rank correlation coefficients, as normality assumptions were not met for one or more variables in each group and task.

In the cognitive task, fatigue was positively correlated with perceived effort in both groups. In the physical task, no significant correlations were observed in the control group. In the long COVID group, fatigue was positively correlated with perceived effort. No other significant correlations were found (Table 9).

**TABLE 9:** Correlations between autonomic function, state fatigue and perceived effort.

Task	Group	HRV – Fatigue	HRV – Effort	Fatigue – Effort
Cognitive	Control (*n* = 21)	0.576 (*P *= 0.00631)	0.388 (*P* = 0.08205)	**0.648 (*P* = 0.00148)**
Cognitive	Long COVID (*n* = 22)	−0.043 (*P* = 0.8505)	0.137 (*P* = 0.545)	**0.723 (*P* ≤ 0.0001)**
Physical	Control (*n* = 12)	0.528 (*P* = 0.0776)	0.068 (*P* = 0.833)	0.497 (*P* = 0.09996)
Physical	Long COVID (*n* = 13)	0.222 (*P* = 0.466)	0.130 (*P* = 0.673)	**0.834 (*P* = 0.00040)**

*Note*: Values are spearman's rho correlation coefficient (*P*‐value). *P*‐values shown in bold indicate statistical significance following Bonferroni correction (*P* < .00417). HRV: the proportion of successive inter‐beat intervals that differed by >50 ms in the final minute of the task. State fatigue: numerical rating scale for fatigue taken at the end of the task. Perceived effort: Borg rating of perceived exertion scale measured at the end of the task. For HRV, participants were excluded if HR could not be clearly identified from the recorded electromyography data. This resulted in smaller sample size than for other analyses. Sample size for each correlation is shown.

**Planned exploratory analysis**: Test–retest reliability for each somatosensory processing measure (gating and attenuation), from the baseline measure from each session (cognitive/physical). To assess the stability of somatosensory processing over time, test–retest reliability was calculated for gating and attenuation measures made at the start of each testing session using the intraclass correlation coefficient (ICC, type 1‐1, absolute agreement). Both gating (ICC = 0.366 [95% CI: 0.159, 0.543], *P* ≤ 0.0001) and attenuation (Intraclass Correlation Coefficient [ICC] = 0.376 [95% CI: 0.169, 0.551], *P* = < 0.0001) showed poor to fair reliability. This suggests that somatosensory processing measures were only moderately stable over time.

## 4 DISCUSSION

The aim of this study was to quantify somatosensory processing in long COVID and examine interactions between somatosensory processing, trait fatigue, fatigability, state fatigue induced by cognitive and physical exertion, autonomic dysfunction, mood, and illness beliefs. We found no differences in somatosensory attenuation or gating between people with long COVID fatigue and controls, and no associations between somatosensory measures and trait fatigue, state fatigue or fatigability. We therefore find no evidence from somatosensory measures to support the sensory attenuation model of fatigue (Kuppuswamy, 2022) in long COVID. Depression and illness beliefs were associated with trait fatigue, supporting the need for psychological impact to be considered within our understanding of fatigue in long COVID.

Participants with long COVID had high levels of trait fatigue, with 64% reporting severe fatigue on the recommended fatigue measure in this population (FAS) (Gorst et al., 2023). Our study sample is therefore consistent with previous studies where long COVID participants report fatigue as a severe and debilitating symptom (Lopez‐Leon et al., 2021; Walker et al., 2023). Compared with controls, individuals with long COVID reported greater trait fatigue across cognitive and physical domains and greater state fatigue throughout the testing sessions.

### 4.1 Somatosensory processing in long COVID

Somatosensory processing was not impaired in our sample of participants with long COVID. The absence of a difference in somatosensory gating at baseline (hypothesis 1; H1) replicates the findings of Baker et al. (2023). Our sample was similar to that of Baker et al. (2023) in terms of sex and age range. However, the interval between acute infection and the first testing session was substantially longer (548–1999 days in comparison to 42–179 days in Baker et al. (2023)). This suggests that somatosensory gating remains stable even in individuals who have experienced long COVID for a much longer duration.

Somatosensory attenuation was also not impaired in our sample of participants with long COVID (H1). This is the first report of somatosensory attenuation in individuals with long COVID, and the absence of any detectable difference is a novel finding. The level of attenuation within our sample (*n* = 1–2) is comparable to values reported in a previous study, indicating that attenuation in our participants falls within the range previously observed in healthy adults when using similar methods (Wolpe et al., 2018). Gating and attenuation are distinct phenomena involved in somatosensory processing (Kilteni & Ehrsson, 2022), reflecting the way our nervous system processes external sensory input during movement (gating) or the sensory consequences of self‐generated actions (attenuation). Our results suggest that neither of these processes is altered in people with long COVID.

A second novel element of this study is the evaluation of somatosensory processing before and after an acute increase in state fatigue. We found no evidence that attenuation or gating was associated with state fatigue: baseline attenuation and gating were not associated with post‐task state fatigue (H2), and the change in somatosensory measures from pre‐ to post‐task was not associated with the change in performance (fatigability) or increases in state fatigue across the task (H3). This further strengthens the interpretation that somatosensory processing is not associated with fatigue in this study sample.

Overall, our findings suggest that neither somatosensory attenuation nor gating is impaired in individuals with long COVID fatigue, nor are they associated with state fatigue experienced following a cognitive or physical task. Planned exploratory analyses revealed low test–retest reliability of these measures, potentially limiting sensitivity to detecting subtle effects. However, the consistency of results across analyses, along with prior findings (Baker et al., 2023) increases confidence that these results reflect preserved somatosensory processing in this population. Accordingly, we found no evidence to support the sensory attenuation model of fatigue (Kuppuswamy, 2017, 2022) in long COVID.

### 4.2 Autonomic system function in long COVID

Long COVID participants had low HRV measured during quiet sitting in the pre‐task assessment of sensory attenuation. This suggests altered autonomic nervous system regulation and is consistent with previous reports of low resting HRV in long COVID (Baker et al., 2023; da Silva et al., 2023). Low HRV is commonly interpreted as reflecting reduced parasympathetic activity and relative sympathetic dominance, and may reflect diminished adaptive cardiovascular control.

However, low resting HRV was not associated with trait fatigue (H4). Planned exploratory analyses also found that HRV measured during task performance was not associated with state fatigue, perceived effort or task‐induced changes in state fatigue. These results indicate that, while autonomic alterations are evident at rest, HRV does not appear to be directly linked to either trait or state fatigue in this cohort.

Previous research has reported lower HR during walking in individuals with long COVID, which was associated with 6MWT distance (Borges et al., 2025). By contrast, the present study found no differences between groups in HR, either at rest or during exertion, and task‐induced HR changes were comparable between long COVID participants and controls. These findings suggest that cardiovascular load does not increase disproportionately during exertion in long COVID.

The absence of group differences in HR during the 6MWT may reflect the relatively submaximal, self‐paced nature of the task, which may not have been sufficiently demanding to reveal abnormalities in cardiovascular responses to exertion. It is possible that differences would become more apparent during higher‐intensity or more prolonged exercise, when physiological reserve is challenged to a greater extent.

Consistent with reduced HRV, individuals with long COVID reported elevated autonomic symptoms on the COMPASS‐31, replicating previous findings (Larsen et al., 2022). However, autonomic symptom burden was not associated with trait fatigue measured with FAS (H4). This contrasts with a separate analysis reported elsewhere (Thomas et al., 2025), which incorporated this sample of individuals with long COVID within a larger sample (*n* = 183) and found that COMPASS‐31 score was associated with total fatigue on the FAS. The fact that an association between autonomic symptoms and fatigue emerged only when these participants were analysed within a larger combined sample suggests that the relationship may be small and detectable only with greater statistical power. Within the present study, limited sample size may have reduced sensitivity to detect this association, despite autonomic dysfunction being a consistent feature of long COVID.

The absence of associations between HRV and trait or state fatigue suggests that autonomic alterations and fatigue in long COVID may represent at least partially dissociable phenomena. While autonomic dysfunction appears to be a common feature of long COVID, it was not associated with individual differences in fatigue or perceived effort during physical or cognitive exertion in the present study. One explanation may be that autonomic dysregulation in long COVID reflects a stable background physiological alteration that is not directly associated with fatigue.

The lack of an association in the present study may suggest a mediated relationship, whereby autonomic dysfunction is associated with fatigue indirectly through other altered processes. If such mediation occurs, direct correlations may not be detectable, particularly in smaller samples. Interpretations should be cautious, as HR and HRV measures were only available in the smaller subsample of participants who participated in this lab‐based study, reducing statistical power to detect relationships.

### 4.3 State fatigue and fatigability in long COVID

Individuals with long COVID reported greater state fatigue than controls at all time points during testing, and greater state fatigue and perceived exertion at all time points during both the cognitive and the physical tasks. Planned exploratory analyses revealed that state fatigue and perceived effort were positively correlated, indicating that participants who felt more fatigued also experienced greater effort. In both tasks (cognitive and physical), individuals with long COVID exhibited greater increases in fatigue and perceived exertion across the duration of the task than controls did. This is consistent with longitudinal data in long COVID showing that both cognitive and physical activities are associated with increased state fatigue (Greenwood et al., 2024). Despite the higher levels of fatigue and perceived effort, and the greater increase in these measures across tasks, the change in task performance was similar across groups. In fact, neither group showed a decline in performance across the tasks, with BIS and walking speed increasing from the start to the end of the cognitive and physical tasks, respectively.

Post‐task state fatigue was not associated with the change in task performance (H2), indicating that greater post‐task fatigue did not correspond to greater performance deterioration. This aligns with previous research showing that fatigue in long COVID is independent of performance decline in isokinetic tasks (Fietsam et al., 2023), and extends this evidence to cognitive and alternative physical tasks. These findings support the observed dissociation between state fatigue and measurable change in performance.

Individuals with long COVID performed worse than controls on the cognitive task. Starting at a lower level of performance in both tasks would mean there was less room for decline, potentially masking differences in fatigability between groups. However, the fact that walking speed and BIS *increased* from the start to the end of the task suggests that the absence of fatigability was not due to a floor effect. Individuals with long COVID also walked a shorter distance than controls during the 6MWT, indicating a lower level of performance in the physical task. The average distance walked in this study (334 m) is similar to that reported in other long COVID samples before rehabilitation (ranging from 358 to 438 m depending on body mass index classification) (Tache‐Codreanu et al., 2024), and similar to that reported in individuals with severe multiple sclerosis (389 m) (Goldman et al., 2008). This is despite differences in the method used to administer the test and supports the use of a self‐paced treadmill to perform the 6MWT as an alternative to overground walking. In future studies, use of a self‐paced treadmill to administer the 6MWT would allow for the measurement of physiological parameters more easily than overground walking, and would allow inclusion of biomechanical indicators of walking performance and fatigability such as the Fatigue Index Kliniken Schmieder (Weich et al., 2022).

These differences in performance may reflect reduced capacity to perform both cognitive and physical tasks, or may reflect the adoption of a pacing strategy, whereby individuals with long COVID consciously or unconsciously regulated their effort to preserve performance over time. Such strategies are commonly reported in chronic fatigue conditions (Casson et al., 2023; Geraghty et al., 2019), and are discussed in more detail below.

### 4.4 Potential mechanisms underlying elevated state fatigue in long COVID

Overall, we found no evidence to support our a priori hypothesis that impaired somatosensory attenuation and gating contribute to elevated state fatigue and perceived effort observed in long COVID. These sensory filtering mechanisms appear intact, and the elevated fatigue therefore likely arises from other processes. Planned exploratory mediation analyses suggest that autonomic function during exertion was not associated with task‐induced fatigue indirectly via somatosensory attenuation or gating in either group. Although some direct effects of autonomic dysfunction during exertion on attenuation or gating were observed, for example in controls during the cognitive task and in long COVID participants during the physical task, these did not translate into changes in fatigue, and no significant indirect effects were detected. Taken together, these results suggest that the elevated fatigue and perceived effort experienced by individuals with long COVID during the physical and cognitive tasks are unlikely to be driven by deficits in somatosensory processing or task‐related autonomic changes.

One interpretation of the observed results is that individuals with long COVID have reduced capacity to perform physical and cognitive tasks, requiring them to engage compensatory mechanisms (motivational, attentional or physiological) to maintain performance throughout the task. These compensatory processes may have increased state fatigue and perceived effort while mitigating measurable performance decrements. This idea of reduced capacity is consistent with evidence that individuals with long COVID have lower oxygen uptake during walking, and this was associated with lower distance walked on a 6MWT (Borges et al., 2025). Similar reductions in capacity have been seen in cognitive tasks including long COVID participants having greater reaction times during cognitive tasks (Zhao et al., 2024) and poorer attention and working memory performance (Boutet et al., 2025) compared to controls. However, any compensatory processes involved in maintaining performance through the task duration were not captured by the measures used in this study, and future research incorporating additional psychological (e.g., motivation, attention) and physiological (e.g., brain activity, muscle recruitment, oxygen consumption) measures during task performance is needed to provide a more complete understanding.

Alternatively, the results observed during the physical and cognitive tasks could be interpreted within the active inference framework, whereby increased fatigue and perceived effort arise from altered interpretation of internal bodily signals rather than from the engagement of compensatory mechanisms required to sustain performance. Within this framework, interoceptive signals related to exertion may be weighted more strongly or interpreted as more threatening, leading to increased fatigue and effort independently of compensatory engagement. This offers a complementary explanation for the observed dissociation between experience and performance. Altered interoception has been theorised to play a role in long COVID symptoms (Burton et al., 2023). If this is altered, the sensation of bodily changes may be amplified, meaning that bodily changes feel more intense. In long COVID, fatigue could be therefore influenced by both physiological changes, that is, inflammation or immune activation, as well as the way the body perceives these changes, potentially explaining between person variability of symptoms seen in long COVID. However, this has not been empirically tested and therefore remains speculative.

Interpretation of the results is also influenced by the likelihood that individuals with long COVID adopted a pacing strategy, consciously or unconsciously regulating their effort to preserve performance over time. Such strategies are common in chronic fatigue conditions (Casson et al., 2023; Geraghty et al., 2019), and could help explain the maintenance of performance. Pacing may reflect an adaptation to reduced capacity, a response to altered processing of interoceptive signals or a combination of both.

These explanations are not mutually exclusive and may interact. Future studies incorporating neural and physiological markers are needed to elucidate specific compensatory mechanisms that support performance during physical and cognitive tasks. Future studies may also evaluate interoceptive processing in long COVID, to support or refute the possibility that this plays a role in the experience of the condition, and should identify if compensatory engagement and/or altered interoceptive processing shape the experience of fatigue. A key strength of this study is that responses to both types of tasks were examined in the same individuals. We observed elevated fatigue and perceived effort in both domains, underscoring the value of this multi‐domain approach, but were not able to identify any indicators of underlying mechanisms. Future work must also look to determine whether any underlying mechanisms are common to both task types, or differ between physical and cognitive demands.

From an applied perspective, these findings suggest that elevated fatigue in long COVID is not necessarily accompanied by immediate reductions in task performance, at least under controlled conditions. This may partly explain why individuals often report that they can ‘push through’ demanding activities in the moment but experience pronounced symptom exacerbation afterward. Our results suggest that physiological measures of task performance should be interpreted with caution, as they may not capture the elevated subjective effort or fatigue experienced by individuals with long COVID.

In our sample of long COVID and control participants, older age was associated with lower state fatigue reported following exertion. While this finding may appear initially counterintuitive, it has been shown that clinical presentations of long COVID symptoms can vary by age (Fain et al., 2025). Our findings may reflect this variation, indicating that the experience of fatigue following a task differs with age. This could also reflect more cautious pacing, a conscious or unconscious strategy to limit increases in post‐task fatigue, with increasing age. This finding highlights the complex nature of fatigue and suggests that age is an important factor that should be considered in interventions for fatigue.

### 4.5 Interacting physiological and psychological factors

Among the long COVID participants, there were high levels of depression, anxiety, threatening illness perceptions, changes in aspects of interoceptive awareness (greater noticing and worrying and less trust of bodily sensations) and disrupted self‐reported autonomic system function. This is consistent with previous reports of this population (Elboraay et al., 2025; Engelmann et al., 2024; Furlanis et al., 2024). Depression and greater perceived illness threats were associated with trait fatigue, but anxiety, autonomic system function, and the increase in state fatigue induced by a physical and a cognitive task were not (H4). The absence of a relation between the acute changes in state fatigue experienced during physical and cognitive tasks and the trait fatigue reported over a longer time scale is consistent with prior work. It is established in individuals with multiple sclerosis that state and trait fatigue do not always correlate (DeLuca, 2024; Heine et al., 2016). Changes in state fatigue may reflect immediate central or cognitive demands, compensatory effort or other task‐specific factors rather than trait characteristics.

We originally hypothesised that autonomic function, illness beliefs and mood would impact fatigue through impaired somatosensory processing (Figure 1). Our results support the hypothesis that autonomic function, illness beliefs, and anxiety and depression are altered in people with long COVID, and that trait fatigue is associated with depression and illness beliefs. However, we did not find evidence to support an association between autonomic function and trait fatigue and did not find evidence that psychological factors are associated with trait fatigue through impaired somatosensory attenuation or gating.

In this study we found that depression and illness beliefs were associated with total fatigue as measured by the FAS. This is consistent with previous reports of associations between depression (Furlanis et al., 2024) and illness perceptions (Bierbauer et al., 2022) and trait fatigue in long COVID. In a separate analysis reported in Thomas et al. (2025), we found that autonomic symptoms were also associated with total fatigue as measured by the FAS. These findings must not be interpreted as causal, and more work is required to determine whether there are directional or shared common mechanisms between these variables. However, our results contribute to the growing evidence of their associations.

We propose combining complementary frameworks, including active inference and the common‐sense model of self‐regulation, to better contextualise the findings of this study and the observed associations between fatigue and psychological impacts. We have already proposed such a model (Thomas et al., 2025). According to this combined approach, if the low resting HRV in this sample reflects autonomic dysfunction, the nervous system's usual reflexes for correcting mismatches between expected and actual sensory input (e.g., adjusting heart rate or blood flow) (Parr et al., 2022) may not function properly, resulting in persistent prediction errors. This combined framework may also help explain the consistent associations between depression and fatigue, as depressive states can alter expectations of bodily sensations, shaping the nervous system's generative model. Persistent prediction errors could further contribute to fatigue, potentially creating a bidirectional relationship. Individual differences in fatigue may arise because some people have stronger prior beliefs or altered interoception, which can amplify bodily sensations and make fatigue feel more intense. However, this has not been tested empirically and therefore remains speculative.

### 4.6 Strengths and limitations

By integrating physiological and psychological approaches, this study combined somatosensory, autonomic and self‐report measures to provide a comprehensive account of fatigue in long COVID. Conduct of the study as a registered report enhances transparency and provides reassurance that all hypotheses, methods and analyses were pre‐specified prior to data collection.

The low level of attrition from the laboratory testing sessions indicates that both cognitive and physical testing were well tolerated and acceptable for this population. Analyses revealed that neither the order of task presentation (which was randomised) nor the session timing (morning vs. afternoon) had significant effects on post‐task fatigue or somatosensory measures, suggesting that these procedural variables did not confound the results.

A battery of validated and widely accepted measures was employed, facilitating comparison with prior research and interpretation within the broader literature. However, with the exception of the BIPQ, which explicitly allows for population‐specific adaptations, none of the measurement tools were designed specifically for use in long COVID populations. Many self‐report measures exhibited scores clustering at the scale maximum (Figure 3), indicating potential ceiling effects and suggesting that these instruments may fail to capture the full range of symptom severity and functional impairment in this population. Factors such as sleep quality, pain, and medication use may have influenced fatigue or physiological measures but were not evaluated.

The cognitive task used in the present study was a shortened (15 min) adaptation of the original 120‐min protocol (Hassan et al., 2024). Preliminary testing conducted prior to the Stage 1 Registered Report demonstrated that this modified version increased fatigue in young healthy participants (Thomas, Pattinson, Bundy et al., 2024), supporting the validity of the shortened task as a fatigue induction procedure. However, in the current study fatigue increased across the task in participants with long COVID, but not in controls. This suggests that, while the shortened task can elicit fatigue, its effects may depend on participant characteristics. The shortened version should be considered an adaptation of the original paradigm, and full equivalence with the longer protocol developed by Hassan et al. (2024) cannot be assumed.

Only acute state fatigue was measured, and longer‐term fluctuations or post‐exertional effects (e.g., post‐exertional symptom exacerbation) were not assessed. While HR and HRV were measured, the inclusion of other markers such as, brain activity, muscle recruitment patterns and oxygen consumption during task performance could provide a more complete picture of physiological function. The observational design means that associations between fatigue, effort, autonomic measures and somatosensory processing cannot establish causality.

### 4.7 Future directions

The sensory attenuation model of fatigue (Kuppuswamy, 2022) considers multiple modalities of sensory input, including visual and auditory input, and their potential associations with fatigue. While the present study finds no evidence for altered somatosensory processing, future research may investigate visual and auditory attenuation in long COVID.

Characterising the mechanisms associated with fatigue in specific populations may be important for identifying meaningful subtypes, particularly in post‐infectious conditions where heterogeneity in clinical presentation is evident. Such mechanistic stratification may help inform targeted interventions, enabling approaches to be better aligned with the processes contributing to fatigue. More broadly, failure to account for any heterogeneity in underlying mechanisms may contribute to variability in treatment response and limit the ability of clinical trials to detect effects. Accordingly, incorporating mechanism‐informed stratification may be important for the design and interpretation of future therapeutic studies.

The absence of an HRV response to challenge in the long COVID group may itself be informative, potentially reflecting reduced autonomic flexibility rather than simply altered resting parasympathetic activity. While speculative, this raises the possibility that dynamic autonomic responses may be relevant for characterising fatigue. However, the mechanisms underlying this pattern cannot be determined from the present data, and the use of HRV reactivity as a phenotyping tool remains to be established.

Future studies should examine psychological (e.g., motivation, attention) and physiological (e.g., brain activity, muscle recruitment, oxygen consumption) mechanisms that may support the maintenance of performance alongside increasing effort and state fatigue in long COVID. Alternative mechanisms of compensatory mechanisms, such as active inference and interoception, have also been proposed where altered interpretation or weighting of internal bodily signals, consistent with interoceptive or active inference frameworks, could provide a framework of how these findings are related to fatigue. These models remain speculative in long COVID and require direct empirical testing.

### 4.8 Conclusion

Our findings confirm that long COVID is associated with elevated trait and state fatigue, emotional distress, and greater autonomic symptoms as measured by the COMPASS‐31, as well as greater increases in perceived effort and state fatigue across both physical and cognitive tasks. These experiences occurred in the absence of any detectable differences in performance decline or somatosensory processing and were not associated with autonomic function. Therefore, we found no evidence to support the sensory attenuation model of fatigue in long COVID, or our a priori hypotheses of associations between somatosensory processing, autonomic function and fatigue.

We have offered two possible interpretations for the observed dissociation between the changes in experience (fatigue, effort) and objective performance over the course of the tasks. One possibility is that individuals with long COVID had a more rapidly declining capacity to perform the physical and cognitive tasks and recruited compensatory mechanisms to maintain performance, resulting in heightened perceived effort and fatigue. An alternative explanation is that the perception of internal bodily demand was amplified in individuals with long COVID, even in the absence of increased load. This is consistent with active inference frameworks, which have been implicated in many conditions but not yet tested empirically in long COVID fatigue. These two explanations are not mutually exclusive, and future studies incorporating a more extensive range of psychological, neural and physiological markers are required to identify the mechanisms driving the experience of fatigue.

## AUTHOR CONTRIBUTIONS

All data collection was performed in the facilities at Cardiff University. Bethan Thomas, Rachael Pattinson, Chris Bundy, and Jennifer Davies contributed to the conception and design of the study. Bethan Thomas and Geoffrey Cunningham collected the data. Bethan Thomas, Geoffrey Cunningham and Jennifer Davies processed the data. Bethan Thomas and Jennifer Davies analysed the data and wrote the first draft of results and discussion. All authors revised the work critically for important intellectual content. All authors have approved the final version of the manuscript, and all authors agree to be accountable for all aspects of the work in ensuring that questions related to the accuracy or integrity of any part of the work are appropriately investigated and resolved. All persons designated as authors qualify for authorship, and all those who qualify for authorship are listed.

## CONFLICT OF INTEREST

None declared.

## GENERATIVE AI STATEMENT

No generative AI tools were used in the preparation of this manuscript.

## Supporting information



Supplementary material 1

Supplementary material 2

Supplementary material 3

Supplementary material 4

Supplementary material 5

Supplementary material 6

Supplementary material 7

Supplementary material 8

## Data Availability

All supplementary material S1–S10 and data supporting the results of this study, including raw experimental data, analysis scripts, and the questionnaire, are openly available at the Open Science Framework repository: https://osf.io/vwy26
