# Supplementary material for: Somatosensory processing in long COVID fatigue and its relations with physiological and psychological factors
Source: Exp Physiol. 2026 Sep 25:10.1113/EP093600. Online ahead of print. doi: 10.1113/EP093600 (PMC13614499; doi:10.1113/EP093600)
Supplement: Supplementary file 1 [file EPH-9999-0-s001.docx]

**Somatosensory processing in long COVID fatigue and its relations with physiological and psychological factors**

**Cognitive task**

This task is completed by participants during the cognitive testing session. It consists of a battery of four different cognitive tasks, each designed to challenge a different aspect of executive function: A-X Continuous Performance Test (AX-CPT), n-back, mental rotation task, and visual search task.

### A-X Continuous Performance Test

In the AX-CPT, participants see a series of four letters consisting of a cue, two distractors, and a probe in each trial. The cue is shown in red and could be any letter other than K or Y. Distractors are shown in white and can be any letter other than A, K, X, or Y. The probe is shown in red and could be any letter other than K or Y. There are four different types of trial. In target trials, A is the cue and X is the probe. The three non-target trials followed a B-X, A-Y, or B-Y cue probe sequence, where B and Y represent any possible letter other than A or X.  Participants are tasked with making a target response when they detect the “AX” sequence (an A cue followed by an X probe), and a non-target response to all other letter sequences (AY trials: an A cue followed by any probe other than X, BX trials: any cue other than A followed by an X probe, and BY trials: any cue other than A followed by any probe other than X). Participants are instructed to press the target button with the middle finger of their right hand as quickly as possible whenever they observed an A cue followed by an X probe, and to press the non-target key with the index finger of the right hand as quickly as possible whenever they observed any other letter pair.

Figure 1 - AX-CPT example

Figure 1 – AX-CPT example. Figure is a timeline of the task. The first flow diagram indicates the timing of the task and what each letter will mean. The second flow diagram is an example of how the task will look to participants.

### N-back

In the n-back task, participants are shown a series of items and are required to indicate whether the current item is the same as the item shown n items previously. We used a 2-back task where participants have to indicate whether the current letter was the same as the letter shown 2 letters ago. Participants respond ‘k’ if it was the same (30% of trials) and ‘d’ if it was not (70% of trials). Trials are presented in a pseudorandom order, where in each 10 trials, three are target trials and seven are non-target trials. Each letter is presented for 2000 ms, followed by 1000 ms of feedback and a 1200 ms interval as in the AX-CPT task (Figure 2). All of the letters are shown in a white font on a grey background at the same height as the letters in the AX-CPT task.

Figure 2 - N-back task example

Figure 2 – N-back task example Figure is a timeline of the task. The first flow diagram indicates the timing of the task and what each letter will mean. The second flow diagram is an example of how the task will look to participants.

### Visual search task

Participants are shown 11 letters on a screen which could be rotated either 0, 90, 180, or 270 degrees. The letters could appear in any space on an invisible 4 x 4 grid. The grid is centred and the size is normalised so that it would leave a border of 25% of the participants’ screen size on all sides. Every trial consists of at least ten letter Ls. Half of the trials are target trials where a letter T is also present in the grid in a random orientation and position (Fig. 3a). The other half of the trials are non-target trials where an additional letter L present (Fig. 3b). Trial order will be pseudorandomised so that in every ten trials, five are target trials and five are non-target trials. Participants are told to press ‘k’ when the T is shown and ‘d’ otherwise. There is no time limit for participants to respond. Once they respond, 1000 ms feedback and a 1200 ms interval are shown as in the AX-CPT and n-back tasks. Letters are presented as in the n-back task.

Figure 3 - Visual search task example

Figure 3 – Visual search task example – Figure 3a and 3b are examples of how the task will look to participants. The first image shows an example where a T is shown, and participants will be required to press ‘k’. The second image is an example of a non-target trial.

### Mental rotation task

In mental rotation tasks, participants are presented with an image of two items and asked to determine whether the items match. One item may be rotated in relation to the other. The left-hand shape is always oriented in the same way, with the right differing by 0, 50, 100, or 150 degrees around a vertical axis (25% of the set differs by each amount). Half of the stimuli show matching shapes (Fig. 4a) and half show non-matching shapes (Fig. 4b). The non-matching shapes are pseudo-mirror images which are made of the same number of cubes and have the same configuration of arms as the matching shapes. Participants will be presented with each stimulus for 7500 ms and required to respond with ‘k’ if the shapes matched and ‘d’ if they did not. As in the other tasks, participants will receive written feedback for 1000 ms, followed by a 1200 ms interval (Fig. 4c).

Figure 4 - Mental rotation task example

Figure 4 – Mental rotation task example - An example of matching shapes is shown in Figure 4a and non-matching shapes in Figure 4b. The timeline of how the task will work is shown in Figure 4c.
