# Supplementary material for: Somatosensory processing in long COVID fatigue and its relations with physiological and psychological factors
Source: Exp Physiol. 2026 Sep 25:10.1113/EP093600. Online ahead of print. doi: 10.1113/EP093600 (PMC13614499; doi:10.1113/EP093600)
Supplement: Supplementary file 2 [file EPH-9999-0-s002.docx]

# Data Handling Procedures

## Online self-report data

Data collected from participant questionnaires were transferred into Excel for analysis in MATLAB and SPSS. The following steps outline data management procedures.

**Standardisation of Data Entry**

- All questionnaire responses were collated into an excel file (Raw_Questionnaires.xlsx)
- Numerical and categorical responses were standardised to ensure consistency across participants.
- Questionnaire responses that used text-based anchors (e.g., *“0 = absolutely no control”*) were reformatted into their corresponding numerical format (e.g., *0*) for the purpose of analysis. These changes were saved in a clean excel file (Raw_Questionnaires_Clean.xlsx).

**Identification of Missing Data**

- The clean dataset (Raw_Questionniares_Clean.xlsx) was reviewed for missing data points across all questionnaires. A total of x missing responses were identified.
- Missing data by questionnaire:
  - CFQ-11: Two participants missing all items. One participant was missing all but Q6.

One participant missing Q1 & Q10, one participant missing Q8, one participant missing Q9.

- FAS: Two participants missing all items. Two missing Q4, one missing Q8.
- MFIS: One participant missing all items, one participant missing Q1 & Q3, one participants missing Q13, one missing Q6.
- BIPQ: Two participants missing Q2. Four participants missing Q4. One participant missing Q5. One participant missing Q7.
- HADS: One participant missing Q3-Q14. One participant missing Q7.
- COMPASS: One participant missing all items. One participant Q3 – Q13, Q15, Q29-Q31. One participant missing Q6 & Q11. One participant missing Q10 – Q12, Q17, Q25. One missing Q6 & Q7. One missing Q6. Eight participants missing Q11. One missing q10. One missing 21 – 23.
- MAIA: Four participants missing all items. One missing Q10,11, Q15, Q16, Q17, Q19-Q25, Q27-Q29, Q31-37. One participant missing Q3. One participant missing Q2. One missing Q11. One missing Q19. One participant missing 17, 22 & 30. One missing q25. One missing 35-37. one missing q13. one missing q28. One missing 23. One missing 34. One missing 33.
- Further inspection of the missing data points revealed that some missing responses were attributable to the questionnaire design and were not true missing data points.
  - COMPASS: Q5 “In the past year, have you ever noticed colour changes in your skin, such as red, white, or purple?” if answered yes follow up question Q6 asked “What parts of your body are affected by these colour changes? (Check all that apply)” in this the only choices were (1) Hands or (2) Feet. If they did not select one of these it would have been classed a missed response. However, this may have been missed as colour changes are on a different body part and so in this instance participants were given 0. These participants were also be given a 0 on the follow up question Q7 on the severity of these symptoms. These were recoded accordingly in the (Raw_Questionnaires_Clean.xlsx) to preserve the logical flow of responses.
  - COMPASS Q10: Asks whether participants experience a particular symptom. If they answered *“No”* to the follow-up question of severity in Q11 becomes irrelevant. Although an option exists on Q11 to select *“I have not had these symptoms”* in this study 12 participants left Q11 empty after initially answering *“No” on Q10*. In this case, a missing response was interpreted as *“I have not had these symptoms”* and recoded accordingly in the (Raw_Questionnaires_Clean.xlsx) Excel file.
  - BIPQ: Q4 asks whether participants believe treatment can help their symptoms. On this question three participants selected not applicable. This is likely due to the lack of treatments for Long COVID at this time. This is classed as a missed response. However there is an option for 0, so in this instance participants will be given a score of 0 to reflect that treatment is not effective.

**Handling of Remaining Missing Data**

After clarifying missing data points arising from questionnaire design, the remaining missing data were reviewed. The handling strategy for each questionnaire is as follows:

- **CFQ-11**
  - No official guidelines exist for handling missing data on the CFQ-11. This study followed that if a participant missed more than 2 questions they were filtered out of the analysis on this questionnaire.
  - Little’s test indicated that data were missing completely at random (MCAR).
  - Missing values for a participant were imputed by calculating the mean value of the item scores present and replacing the missing values with that score.
- **FAS**
  - No official guidelines exist for handling missing data on the CFQ-11. This study followed that if a participant missed more than 2 questions they were filtered out of the analysis on this questionnaire.
  - Little’s test indicated that data were missing completely at random (MCAR).
  - Missing values for a participant were imputed by calculating the mean value of the item scores present and replacing the missing values with that score.
- **MFIS**
  - The MFIS explicitly instructs participants to answer every question, if one or more responses were missing, the total and subscale scores were not calculated.
- **BIPQ**
  - No official guidelines exist for handling missing data on the BIPQ. This study followed that if a participant had missed more than 2 questions they were filtered out of the analysis on this questionnaire.
  - Little’s test indicated that data were MCAR.
  - Missing values for a participant were imputed by calculating the mean value of the item scores present and replacing the missing values with that score.
- **HADS**
  - No official guidelines exist for handling missing data on the HADS. This study followed recommendations by (Bell et al., 2016), using the half-rule (mean substitution if at least half of subscale items are present).
  - Little’s test indicated that data were missing completely at random (MCAR).
- **COMPASS**
  - No official guidelines exist for handling missing data on the COMPASS. This study followed that if at least half of the subscale items were present, items for that subscale would be imputed using mean substitution.
  - Littles test indicated that data were MCAR.
- **MAIA**
  - No official guidelines exist for handling missing data on the MAIA. This study followed that if at least half of the subscale items were present, items for that subscale would be imputed using mean substitution.
  - Littles test indicated that data were MCAR.

Once all procedures were completed, scores were calculated according to each scale’s instructions. The final scores for each participant were then compiled into a new Excel file (Analysed_Questionnaires.xlsx)

## In person testing sessions

Two sessions (one cognitive, one physical) were stopped early at the participant’s request, both were individuals with long COVID.

The participant that stopped during the cognitive task did not finish session due to the level of fatigue they experienced during and following the cognitive task. The session ended following the post attenuation measure, and therefore the post gating measure was not completed. This was the participants first session; they did return for and fully complete the physical session.

The participant that stopped during the physical task did not finish due to the level of fatigue they experienced during and following the 6 minute walk. The session ended following the 6 minute walk, therefore the post attenuation and gating measure was not completed. This was the participants first session; they did return for and fully complete the cognitive session.

## References

Bell, M. L., Fairclough, D. L., Fiero, M. H., & Butow, P. N. (2016). Handling missing items in the Hospital Anxiety and Depression Scale (HADS): a simulation study. *BMC Res Notes*, *9*(1), 479. <https://doi.org/10.1186/s13104-016-2284-z>
