# Supplementary material for: Somatosensory processing in long COVID fatigue and its relations with physiological and psychological factors
Source: Exp Physiol. 2026 Sep 25:10.1113/EP093600. Online ahead of print. doi: 10.1113/EP093600 (PMC13614499; doi:10.1113/EP093600)
Supplement: Supplementary file 4 [file EPH-9999-0-s005.docx]

Somatosensory processing in long COVID fatigue and its relations with physiological and psychological factors

Data from participants that completed the self-report measures only

**Table 1**

Characteristics of study sample that completed the self-report measures only

|  |  | Control  (n=11) | Long COVID  (n=27) |
| --- | --- | --- | --- |
| Age (Years) | - | 36 [24.0–55.8] | 49 [42.0–56.8] |
| Sex | Female | 6 (54.6) | 20 (74.1) |
|  | Male | 4 (36.4) | 7 (25.9) |
|  | Other | 1 (9.1) | 0 (0) |
| Ethnicity | Asian | 4 (36.4) | 1 (3.7) |
|  | Black | 0 (0) | 1 (3.7) |
|  | Mixed or multiple | 1 (9.1) | 1 (3.7) |
|  | Other | 0 (0) | 1 (3.7) |
|  | Prefer not to say | 1 (9.1) | 0 (0) |
|  | White | 5 (45.5) | 23 (85.2) |
| Test for COVID-19 infection | Clinical diagnosis | 0 (0) | 9 (33.3) |
|  | PCR | 1 (9.1) | 5 (18.5) |
|  | LFT | 4 (36.4) | 13 (48.2) |
|  | Other | 1 (9.1) | 0 (0) |
|  | No COVID-19 infection | 5 (45.5) | 0 (0) |

*Values are median [interquartile range] or n (%). The equivalent information for all participants who completed the in person testing sessions is provided in the manuscript, Table 2. PCR = polymerase chain reaction test; LFT = lateral flow test.*

Figure 1 - Distribution of scores across all measures used to evaluate fatigue severity (top row), impact of fatigue on functioning (second row), mood (third row), autonomic symptoms (third row), interoceptive sensibility (fourth and fifth row) and illness perceptions (bottom row) for control (blue) and long COVID (orange) participants who only completed the self-report measures and the in person testing sessions. The y axes are scaled to show the full range of possible scores on each measure. Boxes show the median and interquartile range, whiskers extend to the most extreme data points within 1.5× the interquartile range. Individual data points are shown by crosses to the left of each box plot. Black dashed lines show clinical severity cutoffs. The equivalent plot for only participants who completed at least one in person testing session is provided in the manuscript, Figure 3. CFQ-11: Chalder Fatigue Questionnaire; FAS: Fatigue Assessment Scale; MFIS: Modified Fatigue Impact Scale; MAIA: Multidimensional Assessment of Interoceptive Awareness; COMPASS: Composite Autonomic Symptom Score; HADS: Hospital Anxiety and Depression Scale; BIPQ: Brief Illness Perception Questionnaire; HADS: Hospital Anxiety and Depression Scale.

**Table 2**

Descriptive statistics of psychometric and clinical measures that completed the self-report measures only

|  |  | Control |  | Long COVID |
| --- | --- | --- | --- | --- |
| *CFQ Bimodal* | 11 | 3.00 [0.00 – 5.50] | 27 | 11.00 [9.75–11.00] |
| *CFQ Likert* | 11 | 13.00 [9.50–16.50] | 25 | 25.00 [23.75–29.00] |
| *CFQ Physical* | 11 | 7.00 [6.00–10.00] | 25 | 16.00 [14.75–19.00) |
| *CFQ Cognitive* | 11 | 5.00 [3.25–7.00] | 25 | 9.00 [7.75–11.00) |
| *FAS* | 11 | 21.00 [18.25–24.25] | 25 | 40.00 [34.25–44.25) |
| *MFIS Total* | 11 | 28.00 [16.50–33.00] | 25 | 68.50 [57.00–76.00) |
| *MFIS Physical* | 11 | 13.00 [5.75–14.75] | 26 | 30.00 [28.00–34.00) |
| *MFIS Cognitive* | 11 | 11.00 (10.00–16.50] | 26 | 31.50 [23.00–35.00) |
| *MFIS Social* | 11 | 3.00 [1.25–3.75] | 26 | 7.00 [6.00–8.00) |
| *HADS Total* | 11 | 8.00 [6.00–14.75] | 27 | 23.00 [16.00–26.75) |
| *HADS – Anxiety* | 11 | 5.00 [3.50–8.50] | 27 | 11.00 [9.00–15.00) |
| *HADS – Depression* | 11 | 4.00 [1.00–6.75] | 27 | 10.00 [7.50–12.75) |
| *BIPQ Total* | – | – | 26 | 64.00 [57.00–69.00) |
| *BIPQ Cognitive* | – | – | 26 | 41.50 [37.00–44.00) |
| *BIPQ Emotional* | – | – | 26 | 16.50 [15.00–18.00) |
| *BIPQ Understanding* | – | – | 26 | 5.50 [4.00–7.00) |
| COMPASS | 10 | 8.91 [5.46–19.45] | 27 | 37.03 [23.59–52.53) |
| Orthostatic Score | 10 | .00 [.00–16.00] | 27 | 20.00 [13.00–28.00) |
| Vasomotor Score | 10 | .00 [.00–.00] | 27 | .00 [.00–1.25] |
| Secretomotor Score | 10 | .00 [.00–2.14] | 27 | 4.29 [.00–8.04] |
| Gastrointestinal Score | 10 | 4.02 [1.79–4.46] | 27 | 8.93 [5.80–11.61] |
| Bladder Score | 10 | .00 [.00–1.11] | 27 | 1.11 [.00–3.06] |
| Pupillomotor Score | 10 | .50 [.00–1.33] | 27 | 2.33 [1.17–3.00] |

Values are median [interquartile range]. The equivalent information for all participants who completed the in person testing sessions is provided in the manuscript, Table 3. CFQ-11: Chalder Fatigue Questionnaire; FAS: Fatigue Assessment Scale; MFIS: Modified Fatigue Impact Scale; MAIA: Multidimensional Assessment of Interoceptive Awareness; COMPASS-31: Composite Autonomic Symptom Score; BIPQ: Brief Illness Perception Questionnaire; HADS: Hospital Anxiety and Depression Scale

**Table 3**

Mann–Whitney U comparing long COVID and controls that completed the self-report measures only

|  | pValue | Z |
| --- | --- | --- |
| *CFQ Bimodal* | 0.110848844 | -1.59438941 |
| *CFQ Likert* | 5.24849E-06 | -4.55460189 |
| *CFQ Physical* | 1.23527E-05 | -4.37126852 |
| *CFQ Cognitive* | 4.98079E-06 | -4.56559532 |
| *FAS* | 0.000691565 | -3.39290152 |
| *MFIS Total* | 0.000136244 | -3.81488995 |
| *MFIS Physical* | 2.24824E-05 | -4.23869351 |
| *MFIS Cognitive* | 6.00796E-06 | -4.52610895 |
| *MFIS Social* | 0.000478425 | -3.49255407 |
| *HADS Total* | 0.002704532 | -2.99946616 |
| *HADS – Anxiety* | 0.008451291 | -2.63348765 |
| *HADS – Depression* | 0.000930372 | -3.3107774 |
| COMPASS | 0.002207204 | -3.06083542 |
| Orthostatic Score | 0.011427484 | -2.52934745 |
| Vasomotor Score | 0.084067985 | -1.72755529 |
| Secretomotor Score | 0.027141589 | -2.20947544 |
| Gastrointestinal Score | 0.000397588 | -3.5416797 |
| Bladder Score | 0.182671654 | -1.33257396 |
| Pupillomotor Score | 0.005163991 | -2.796622 |

The equivalent information for all participants who completed at least one in person testing session is provided in the manuscript. CFQ-11: Chalder Fatigue Questionnaire; FAS: Fatigue Assessment Scale; MFIS: Modified Fatigue Impact Scale; MAIA: Multidimensional Assessment of Interoceptive Awareness; COMPASS: Composite Autonomic Symptom Score; HADS: Hospital Anxiety and Depression Scale; BIPQ: Brief Illness Perception Questionnaire; HADS: Hospital Anxiety and Depression Scale

**Table 4**

*Results of linear mixed-effects model predicting change in somatosensory attenuation intercept*

| **Predictor** | **Estimate (b)** | **SE** | **t(150)** | **p** | **95% CI Lower** | **95% CI Upper** |
| --- | --- | --- | --- | --- | --- | --- |
| Intercept | 0.215 | 0.624 | 0.344 | .731 | −1.019 | 1.448 |
| Group (long COVID) | 0.137 | 0.693 | 0.197 | .844 | −1.233 | 1.507 |
| Task (Physical) | −0.675 | 0.708 | −0.954 | .342 | −2.074 | 0.723 |
| Change in fatigue | 0.015 | 0.027 | 0.568 | .571 | −0.038 | 0.068 |
| Performance fatigability | −0.038 | 0.317 | −0.120 | .905 | −0.664 | 0.588 |

**Table 5**

*Results of linear mixed-effects model predicting change in somatosensory attenuation slope*

| **Predictor** | **Estimate (b)** | **SE** | **t(150)** | **p** | **95% CI Lower** | **95% CI Upper** |
| --- | --- | --- | --- | --- | --- | --- |
| Intercept | 0.004 | 0.117 | 0.038 | .970 | −0.227 | 0.236 |
| Group (long COVID) | −0.023 | 0.130 | −0.179 | .858 | −0.280 | 0.234 |
| Task (Physical) | 0.114 | 0.133 | 0.859 | .392 | −0.148 | 0.376 |
| Change in fatigue | −0.003 | 0.005 | −0.646 | .520 | −0.013 | 0.007 |
| Performance fatigability | −0.007 | 0.059 | −0.124 | .901 | −0.125 | 0.110 |

**Table 6**

Effect of autonomic function on change in state fatigue during the cognitive task via somatosensory attenuation

| **Group** | **N** | **a (X→M)** | **b (M→Y)** | **c′ (Direct)** | **c (Total)** | **ab (Indirect)** |
| --- | --- | --- | --- | --- | --- | --- |
| Controls | 21 | .023 (.094) | –1.127 (.453) | **.283 (.036)** | **.257 (.034)** | –.025 (.823) |
| Long COVID | 22 | –.004 (.986) | .428 (.809) | .309 (.158) | .308 (.109) | –.002 (.867) |

B coefficients (p-values). X = Autonomic Function measured by HRV TP 4; M = Attenuation or Gating; Y = Change in state fatigue. c′ = direct effect of X on Y, c = total effect, ab = indirect effect. N = number of participants. Significant effects (p < .05) are in bold.

**Table 7**

Effect of autonomic function on change in state fatigue during the cognitive task via somatosensory gating

| **Group** | **N** | | **a (X→M)** | | **b (M→Y)** | | **c′ (Direct)** | | **c (Total)** | | **ab (Indirect)** | |
| --- | --- | --- | --- | --- | --- | --- | --- | --- | --- | --- | --- | --- |
| Controls | | 20 | | –.002 (.550) | | –1.131 (.901) | | .252 (.067) | | **.254 (.048)** | | .003 (.600) |
| Long COVID | | 18 | | .031 (.128) | | –1.620 (.473) | | .380 (.105) | | .329 (.126) | | –.051 (.883) |

B coefficients (p-values). X = Autonomic Function measured by HRV TP 4; M = Attenuation or Gating; Y = Change in state fatigue. c′ = direct effect of X on Y, c = total effect, ab = indirect effect. N = number of participants. Significant effects (p < .05) are in bold.

**Table 8**

Effect of autonomic function on change in state fatigue during the cognitive task via somatosensory attenuation

| **Group** | **N** | **a (X→M)** | **b (M→Y)** | **c′ (Direct)** | **c (Total)** | **ab (Indirect)** |
| --- | --- | --- | --- | --- | --- | --- |
| Controls | 12 | 0.046 (.249) | –2.417 (.842) | 0.814 (.277) | 0.702 (.107) | –0.112 (.956) |
| Long COVID | 13 | **–0.078 (.007)** | 2.710 (.500) | –0.029 (.816) | –0.241 (.448) | –0.212 (.636) |

B coefficients (p-values). X = Autonomic Function measured by HRV TP 4; M = Attenuation or Gating; Y = Change in state fatigue. c′ = direct effect of X on Y, c = total effect, ab = indirect effect. N = number of participants. Significant effects (p < .05) are in bold.

**Table 9**

Effect of autonomic function on change in state fatigue during the cognitive task via somatosensory gating

| **Group** | **N** | **a (X→M)** | **b (M→Y)** | **c′ (Direct)** | **c (Total)** | **ab (Indirect)** |
| --- | --- | --- | --- | --- | --- | --- |
| Controls | 12 | **0.013 (.032)** | 35.610 (.219) | 0.234 (.680) | 0.702 (.107) | 0.468 (.297) |
| Long COVID | 13 | 0.038 (.075) | 0.710 (.847) | –0.268 (.525) | –0.241 (.383) | 0.027 (.603) |

B coefficients (p-values). X = Autonomic Function measured by HRV TP 4; M = Attenuation or Gating; Y = Change in state fatigue. c′ = direct effect of X on Y, c = total effect, ab = indirect effect. N = number of participants. Significant effects (p < .05) are in bold.
